# Enhancer RNA transcription near segmentation gene enhancers can be analyzed *in situ* using FISH

**DOI:** 10.1093/g3journal/jkag159

**Published:** 2026-06-19

**Authors:** Christine Mau, Benjamin Schmid, Ezzat El-Sherif

**Affiliations:** Department of Evolutionary Developmental Genetics, Georg-August-University Göttingen, Justus-von-Liebig-Weg 11, Göttingen 37077, Lower Saxony, Germany; FAU Competence Centre Optical Imaging Centre Erlangen, Cauerstr. 3, Erlangen 91058, Bavaria, Germany; School of Integrative Biological and Chemical Sciences (SIBCS), The University of Texas Rio Grande Valley, Science Building, 1201 W. University Dr., Edinburg, TX 78541, United States

**Keywords:** enhancer RNA (eRNA), Drosophila melanogaster, *FISH/HCR imaging*, MS2-MCP live imaging, chromatin insulators, transcriptional bursting

## Abstract

Enhancer RNAs (eRNAs) are non-coding transcripts produced at enhancer regions, which appear to be involved in transcriptional regulation. Up to date, these have been primarily investigated using labor- and cost-intensive genomic techniques. However, the precise mechanisms by which eRNA transcription or eRNA transcripts themselves mediate transcriptional regulation remain unclear. Here, we present a novel experimental approach that allows us to analyze characteristics of eRNA transcription in fixed and live whole *Drosophila melanogaster* embryos. We employ the anterior–posterior patterning genes as a model system to investigate the dynamics of eRNA expression, utilizing an imaging-based approach. We combined high-sensitivity fluorescence *in situ* hybridization chain reaction with high-resolution confocal microscopy to detect eRNA and mRNA molecules. Through this experimental assay, we identified foci of elevated transcriptional activity that generate eRNA transcripts correlated with mRNA production at the same gene locus. We could show that this eRNA transcription is independent of promoter activity. Additionally, we demonstrate that insulators can influence eRNA transcription, resulting in loss of eRNA transcription. Moreover, we observe that eRNAs can originate both within classical enhancer regions and outside of them, including from foreign bacterial sequences when these are placed near enhancer sequences, underscoring the strong influence of local regulatory context on eRNA initiation. In live embryos using MS2-MCP live imaging, our analysis of insulators showed a modest reduction in mRNA burst intensity accompanied by a slight increase in burst frequency. Overall, our imaging-based approach offers a novel platform for dissecting enhancer–eRNA interactions and could be adapted for wider applications.

## Graphical Abstract

**Graphical Abstract:**
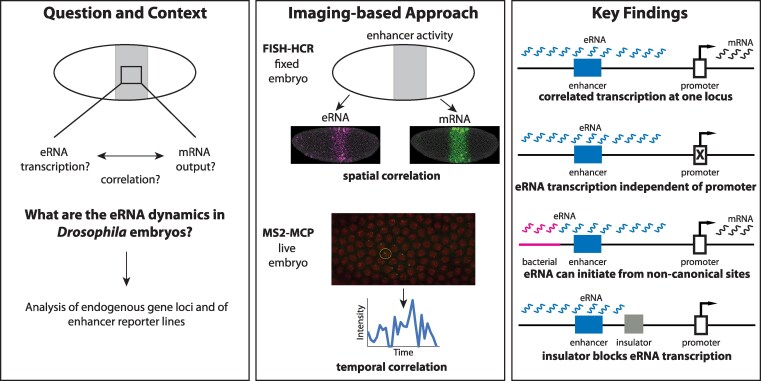

## Introduction

In higher eukaryotes, enhancers are regulatory DNA elements that do not directly activate gene expression but instead modulate the activity of promoters, which function as molecular valves to recruit RNA Polymerase II (Pol II) and initiate mRNA synthesis (Lenhard et al. 2012; Ryu et al. 2024). Despite extensive study, a comprehensive model explaining how enhancers control gene activity and determine transcriptional output remains elusive (Li et al. 2023; Ryu et al. 2024; Zhang et al. 2024).

Recent discoveries, however, provide new avenues for understanding enhancer function. First, transcription is now recognized to occur in bursts, with gene promoters alternating between active and inactive states, resulting in episodic mRNA production. Although enhancers appear to influence transcriptional bursting, the underlying mechanisms remain unclear (Fukaya et al. 2016; Zhang et al. 2024). Second, Pol II molecules transiently form clusters at promoters to initiate transcription, but these clusters dissipate rapidly, even as transcription continues (Cisse et al. 2013; Cho et al. 2016; Pancholi et al. 2021; Ryu et al. 2024). The origin of additional Pol II molecules required for ongoing transcription is not yet understood. Third, transcription is not restricted to promoters; it also occurs at enhancer regions and intervening sequences, producing short, unstable, non-coding transcripts known as enhancer RNAs (eRNAs) (Tuan et al. 1992; Ashe et al. 1997; De Santa et al. 2010; Kim et al. 2010; Carullo et al. 2020; Qiu et al. 2026). Several studies have shown that eRNA transcription can influence gene activity (Kaikkonen et al. 2013; Li et al. 2013; Racko et al. 2018), yet the precise mechanisms remain unresolved (Qiu et al. 2026; Wang et al. 2026). It has been proposed that either the process of eRNA transcription or the eRNA molecules themselves—or both—may mediate transcriptional regulation (Qiu et al. 2026). Fourth, gene promoters not only loop to interact with enhancers, as described in the classical looping model (Splinter et al. 2006; Wendt et al. 2008), but also scan and bind to other DNA sequences in the vicinity and beyond, potentially within dynamic transcriptional condensates (Pancholi et al. 2021; Ryu et al. 2024). How these observations integrate into a unified model of transcriptional regulation is still unclear. Transcriptional regulation is highly dynamic, especially during development (Levine and Davidson 2005), when gene expression patterns change rapidly to guide cell fate decisions. This dynamic nature makes developmental systems an excellent model for studying the principles of transcriptional control.

Several strategies have been employed to investigate the role of eRNA transcription in gene regulation. Genomics-based methods such as global run-on sequencing (GRO-seq), precision nuclear run-on sequencing (PRO-seq), and cap analysis gene expression (CAGE) have enabled genome-wide analysis of nascent transcripts (Carullo et al. 2020). However, these bulk assays average the behavior of millions of cells, limiting their ability to resolve the spatiotemporal dynamics of eRNA transcription. RNA interference techniques—including short hairpin RNAs (shRNAs), small interfering RNAs (siRNAs), and locked nucleic acids (LNAs)—have been used to knock down eRNA transcripts in cell culture, providing insight into their cellular roles (Melo et al. 2013; Hsieh et al. 2014; Pnueli et al. 2015). Nonetheless, cell culture systems may not fully capture the complexity of eRNA function during embryonic development. In contrast, fluorescent RNA *in situ* hybridization chain reaction (FISH HCR) enables the visualization of eRNA transcriptional dynamics and their relationship to gene transcripts at the single-cell level and was shown in cell culture (Shibayama et al. 2017).

In this study, we implemented an imaging-based approach using FISH to investigate the characteristics of eRNA transcription during development. We selected the early patterning of the anterior–posterior (AP) axis as a model system, focusing on the well-characterized AP patterning genes of the fruit fly *Drosophila melanogaster* (*Drosophila*) (Schroeder et al. 2004; Schroeder et al. 2011). This system offers several advantages: (1) a wealth of characterized patterning enhancers and (2) the blastoderm embryo's single layer of nuclei, which facilitates imaging at single-cell resolution.

We concentrated our analysis on enhancers of gap and pair-rule genes, which respectively define the early anterior–posterior axis and the periodic segmentation pattern in *Drosophila* embryos. Using precisely staged embryos, we investigated the endogenous dynamics of eRNA expression at native genomic loci. To further dissect eRNA activity, we developed an enhancer reporter system that allowed controlled assessment of eRNA transcription—both in fixed embryos via in situ HCR and in live embryos using an MS2 reporter assay. Our results show that eRNAs are transcribed not only from active enhancer regions but also from adjacent sequences, even when these are replaced by foreign bacterial sequences. Notably, eRNA transcription displays spatial and temporal patterns that closely parallel those of their associated genes.

## Material and methods

### Fly culture

*Drosophila melanogaster* were maintained in standard vials containing *Drosophila* food prepared as follows (for 10 L): 12.5 L water, 53.2 g agar, 900 g cornmeal, 600 g sugar beet syrup, 150 g dry yeast, 30 g Nipagin, 120 mL 70% ethanol, and 50 mL propionic acid. Flies were kept at 18 °C for stock maintenance. For mating and egg collection, flies were transferred to 25 °C.

### Enhancer reporter constructs

All enhancer reporter constructs were generated using the pbPHi plasmid, which contains a mini-white marker, a minimal *eve* promoter (−50 bp to +50 bp relative to the *eve* transcription start site), a 24xMS2-*yellow* cassette, and an attB site (Bothma et al. 2014). Enhancer regions (Small and Levine 1991; Fujioka et al. 1995, 1999; Small et al. 1996), the su(Hw) insulator (Geyer and Corces 1992; Cai and Levine 1995), intergenic regions, and pbPHi backbone regions were PCR-amplified and inserted into the linearized pbPHi plasmid using In-Fusion cloning (Takara Bio Europe AB). Plasmid linearization was performed with NotI or NotI + BamHI to excise the minimal *eve* promoter as required. Primer sequences are listed in Supplementary Table 1.

### *Drosophila* transgenesis

Enhancer reporter constructs were integrated into chromosome III of the Vk33 strain (RRID:BDSC_9750) via PhiC31 integrase-mediated site-specific transgenesis (BestGene Inc.). Transgenic flies were identified by the mini-white marker and crossed to establish homozygous lines for each enhancer reporter construct.

### Egg collections

For embryo collections, adult flies were placed in cages with agar apple juice plates (2.375% w/v agar, 2.5% w/v sucrose, 25% v/v apple juice) and kept at 25 °C for the desired collection period.

### *In situ* hybridization and imaging of fixed embryos

*In situ* hybridization was performed using the third-generation hybridization chain reaction (HCR v3.0; Choi et al. 2018). Probe sets and hairpins were obtained from Molecular Instruments (see Supplementary Table 2 for lot numbers). Imaging was conducted using a Leica TCS SP5 II confocal laser scanning microscope with 20× or 63× objectives at 2,048 × 1,024 resolution.

### Live imaging

MCP-GFP and Histone-RFP expressing females (Garcia et al. 2013) were crossed with males carrying either the 3&7&2 enhancer > MS2-*yellow* or 3&7-su(Hw) upstream-2 enhancer > MS2-*yellow* constructs. Eggs were collected over 1 h and incubated for an additional 30 min at 25 °C. Embryos were dechorionated in bleach for 1 min 40 s, mounted using the hanging drop method, and covered with halocarbon oil 700 (Sigma) and a membrane to prevent desiccation. Time-lapse imaging was performed on a Leica SP5 II confocal microscope at 21 °C, with 20 planes (z-step size: 0.45 µm) captured every 2 min for approximately 1 h using a 63× objective (512 × 256 resolution). Maximum Z-projections were generated in Fiji (Schindelin et al. 2012) to create unprocessed videos (Supplementary Videos 1 and 2).

### Image processing

Nuclei were detected by Gaussian blurring, thresholding and applying a watershed algorithm. Transcription sites were detected as local maxima in 3D after applying a Difference-of-Gaussian filter. Transcription sites were assigned to the closest nucleus (Supplementary File 1, Program 1). Graphical representation of embryos and AP plots of embryos were generated in Matlab by identifying and quantifying nuclear puncta by extracting nuclei and associating puncta intensities with their respective nuclei. Fluorescence along the DV axis was summed and normalized to generate AP distribution plots (Supplementary File 1, Program 2). For transcription landscape profiling, data of several embryos are plotted on a space axis using Matlab (Supplementary File 1, Program 3).

For bursting analysis of live imaging videos, fluorescence time traces from individual nuclei were generated manually in Fiji. Nuclei traces and burst analysis was performed with Matlab. For each nucleus, bursts were identified as periods when the signal exceeds 5% of the maximum trace intensity. Mean burst amplitude is determined by averaging the maximum signal intensities within each burst. Mean intensity corresponds to the average signal across the entire trace (Supplementary File 1, Program 4).

## Results

### Characterization of eRNA transcriptional properties in the endogenous gene locus

**Fig. 1.**
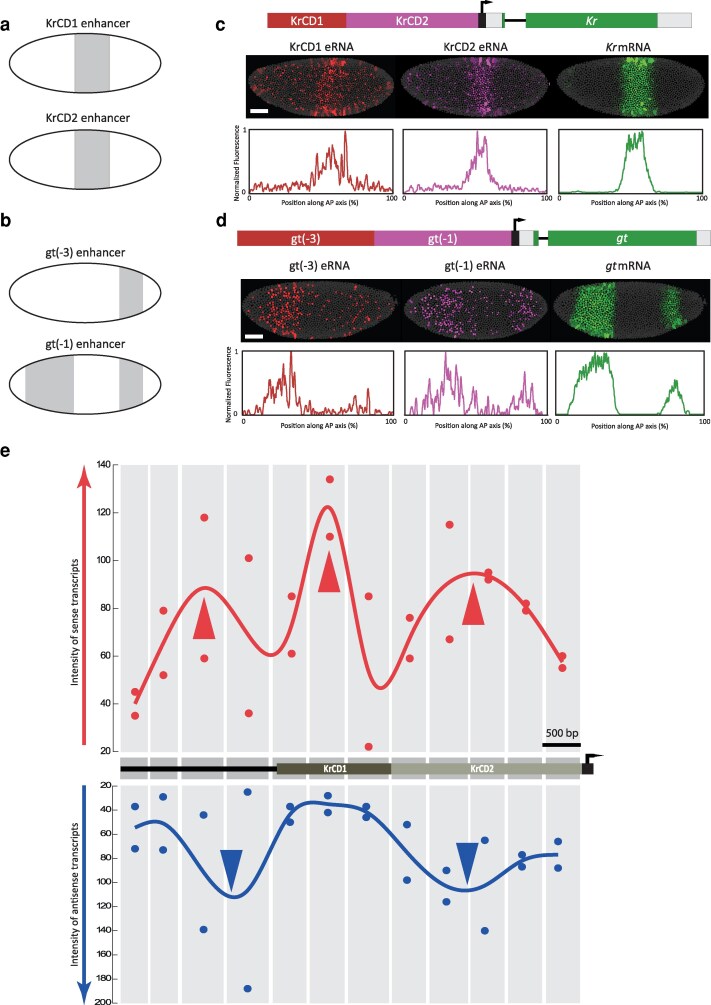
Spatial expression of eRNAs from gap gene enhancers. *Kr* locus and *gt* locus are shown. a, b) Schematics illustrate the activity patterns of selected enhancer regions in the blastoderm embryo. a) The KrCD1 and KrCD2 enhancers drive expression in the central domain. b) The gt(−3) enhancer is active in the posterior domain, whereas the gt(−1) enhancer drives expression in both anterior and posterior domains. c, d) eRNA (red and purple) and mRNA (green) transcripts were visualized together in the endogenous gap gene locus in whole *Drosophila* embryos at nuclear cycle 14 (NC14) using *in situ* HCR staining. Nuclei are shown in gray. Fluorescence signals along the dorsal–ventral axis were summed and normalized to the maximum intensity to generate eRNA distribution profiles along the anterior–posterior (AP) axis (position in %). c) eRNA transcripts from the KrCD1 and KrCD2 enhancers are detected in the central AP domain, overlapping with the *Kr* mRNA expression region. d) *gt* mRNA forms anterior and posterior domains, with eRNA from the gt(−3) and gt(−1) enhancers detected at similar AP positions. Anterior is left, dorsal is up. Scale bars: 50 µm. e) High-resolution mapping of eRNA transcription along the upstream sequence of the endogenous *Kr* locus. A 6-kb upstream region of the endogenous *Kr* locus containing KrCD1 and KrCD2 enhancers was analyzed. eRNA transcripts were visualized by *in situ* HCR staining at NC13. Sense (red) and antisense (blue) eRNA transcripts were detected using 4 to 5 probes spanning ∼500 bp windows. Two embryos were imaged per window. High-resolution confocal imaging was used to detect *Kr* mRNA and eRNA transcription foci. The y-axis shows the intensity of eRNA foci overlapping with *Kr* mRNA foci. Three maxima of sense eRNA—one upstream of KrCD1, one in KrCD1, and one in KrCD2—and two maxima of antisense eRNA—one upstream of KrCD1 and one in KrCD2—were observed (arrow heads).

To investigate the properties of eRNA transcription, we focused on the early anterior–posterior (AP) patterning genes *Krüppel* (*Kr*) and *giant* (*gt*). For *Kr*, we analyzed the enhancers KrCD1 (−3,948 bp to −2,435 bp upstream of the *Kr* transcription start) and KrCD2 (−2,605 bp to −121), both of which are active in a central domain along the embryo's AP axis (Hoch et al. 1990; El-Sherif and Levine 2016) (Fig. 1a). For *gt*, we examined the enhancers gt(−1) [−1,284 bp to −45 bp upstream of the *gt* transcription start] and gt(−3) [−2,500 bp to −1,291 bp]: gt(−1) is active in both, anterior and posterior domains, whereas gt(−3) activity is restricted to the posterior domain (Schroeder et al. 2004) (Fig. 1b). All analyses were performed in *Drosophila* embryos at nuclear cycle 14 (NC14), corresponding to blastoderm cellularization approximately 2 h 10 min to 3 h after egg laying (Foe et al. 1993).

To visualize the low-abundance eRNA transcripts, we employed high-sensitivity fluorescence *in situ* hybridization chain reaction (HCR) staining (Choi et al. 2018). This method enables multiplexed detection (up to five RNA targets simultaneously), quantitative analysis at single-molecule resolution, and sensitive identification of low-abundance RNAs through automatic background suppression. Here, we stained one mRNA and two eRNAs in the same embryo, allowing us to precisely study their relationship. HCR staining was combined with high-resolution confocal microscopy. For HCR staining, probes were uniformly distributed over the assessed region, covering about 50 bp per probe (for probe LOT numbers see Supplementary Table 2).

Consistent with previous reports, eRNA expression was predominantly nuclear. For the KrCD1 and KrCD2 enhancers, eRNA transcripts were detected in a central domain of the embryo, mirroring the expression pattern of *Kr* mRNA (Fig. 1c, for representative raw image see Supplementary Fig. 1a). Similarly, eRNA transcripts from gt(−1) and gt(−3) enhancers were enriched in anterior and posterior domains, closely resembling the *gt* mRNA expression pattern (Fig. 1d, for representative raw image see Supplementary Fig. 1b). Notably, gt(−3) eRNA transcripts were strongly expressed in the anterior domain, despite enhancer activity being confined to the posterior (Schroeder et al. 2004).

We compared our results with publicly available genomic datasets for *Kr* and *gt* (Supplementary Fig. 2a+b; Tracks were visualized using the UCSC Genome Browser [Casper et al. 2026]). PRO-seq data from Hunt et al. (2024) (GEO accession: GSM6454906), generated from embryos at NC7–9 (60 to 80 min after egg lay, AEL), were used to visualize regions of active transcription in both sense and antisense directions. At the *Kr* locus, no peaks were detected in the sense direction, whereas the antisense signal showed peaks overlapping with the KrCD1 and KrCD2 enhancer regions, which also displayed eRNA transcription in our FISH analysis. At the *gt* locus, no antisense peaks were observed, but several sense peaks were present in the gt(−3) and gt(−1) enhancer regions. Notably, a strong sense peak was observed in the gt(−3) enhancer region, which may account for the presence of eRNA transcripts in the anterior region of the embryo (compare Fig. 1d). This anterior eRNA transcription may repress gt(−3) enhancer activity in the anterior domain. Such repression of enhancer activity by transcription through the enhancer has previously been reported (Erokhin et al. 2013).

ChIP-seq data from Harrison et al. (2011) (GEO accession: GSE30757), generated from embryos at NC13 (2 h AEL) and NC14 (3 h AEL), were used to visualize genomic regions bound by the pioneer factor Zelda (Zld), which is required for zygotic genome activation. Zld peaks coincided with the eRNA peaks detected by FISH in the KrCD1 and KrCD2 enhancer regions, as well as the gt(−1) and gt(−3) enhancer regions. ATAC-seq data from Soluri et al. (2020) (GEO accession: GSE141538), generated from embryos at NC14 + 12 and NC14 + 72, were used to visualize open chromatin regions that may be permissive for eRNA transcription. These ATAC-seq peaks also overlapped with eRNA transcription in the KrCD1, KrCD2, gt(−1), and gt(−3) enhancer regions.

These observations demonstrate that our FISH-based approached can be used to detect eRNA transcription in a reliable manner. Moreover, eRNA transcription is spatially correlated with mRNA expression of the corresponding gene. The activity of gt(−3) enhancer eRNA transcription at the anterior was surprising in contrast to the reported gt(−3) activity at the posterior, raising questions about the broader landscape of eRNA transcription within gene loci.

### Profiling the landscape of eRNA transcription within the gene locus

Given the observed spatial correlation between eRNA and mRNA transcription, we next sought to systematically profile the abundance of eRNA transcription across a gene locus. Two possible configurations might be expected: (i) eRNA transcripts may be uniformly expressed throughout the gene locus or (ii) eRNA transcripts may arise from discrete, defined regions within the locus.

To this end, we analyzed the 6 kb upstream region of the *Kr* locus, which encompasses the KrCD1 and KrCD2 enhancers. For *in situ* HCR staining, we designed detection windows of 500 bp to map eRNA transcriptional activity at high resolution. As previous studies have demonstrated that eRNAs are frequently bidirectionally transcribed (Melgar et al. 2011; Hah et al. 2013), we probed for both, sense and antisense eRNA transcripts. Sense and antisense probes were alternating for detection of sense and antisense eRNA transcripts in the same embryo (4 to 5 probes per detection window for sense and antisense, respectively).

eRNA distribution was analyzed at nuclear cycle 13 (NC13; syncytial blastoderm, approximately 1 h 50 min to 2 h 10 min after egg laying [Blythe and Wieschaus 2015; Foe et al. 1993]), a developmental stage at which both, mRNA and eRNA transcriptional foci, are readily detectable.

High-resolution confocal imaging was used to detect *Kr* mRNA and eRNA transcription foci. The intensity of eRNA foci overlapping with *Kr* mRNA foci was analyzed along the 6 kb region. Our high-resolution mapping revealed hotspots of eRNA transcription for both, sense and antisense probes (Fig. 1e, arrow heads). We identified several prominent peaks of eRNA transcription: one upstream of the KrCD1 enhancer (sense and antisense eRNA signal), one in the KrCD1 enhancer (sense eRNA signal), and a third one in the middle of the KrCD2 enhancer (sense and antisense eRNA signal) suggesting that these regions serve as sources of eRNA production within the *Kr* locus. In addition, distinct regions within the KrCD1 and KrCD2 enhancers exhibited local minima in eRNA signal.

We compared the obtained landscape profile of eRNA transcription with some publicly available genomic datasets (Supplementary Fig. 2a) and found that they show a similar overall trend, since peaks found by our FISH-based approach coincide with peaks found by PRO-seq, Zld ChIP-seq, and ATAC-seq. Together, these findings validate our FISH-based approach for analyzing eRNA transcription, as the results are consistent with previous sequencing-based studies. Importantly, this approach allows visualization of the eRNA transcription landscape at any gene locus of interest in whole embryos. Because gene regulation is strongly influenced by developmental stage, spatial patterning, and maternal/zygotic context, our method provides the advantage of analyzing these features in intact embryos, which is not easily achievable with sequencing-based approaches.

Collectively, these results indicate that eRNA transcription is not uniformly distributed but instead arises from discrete hotspots within the gene locus. The spatial organization of these hotspots may be shaped by the occupancy of transcription factors and the transcriptional machinery at enhancers and promoters.

### Establishing an enhancer reporter system to study eRNA transcriptional dynamics

Our analysis of eRNA transcription at endogenous gene loci revealed that eRNA synthesis in the embryo occurs in spatial patterns closely mirroring those of mRNA expression. However, in the endogenous context, eRNA transcription may be influenced by the entire regulatory landscape of the genomic locus, which complicates the interpretation of individual enhancer activities. To overcome this limitation, we developed a *Drosophila* enhancer reporter system that enables visualization of eRNA transcription in a controlled and reproducible setting outside of the endogenous locus.

**Fig. 2.**
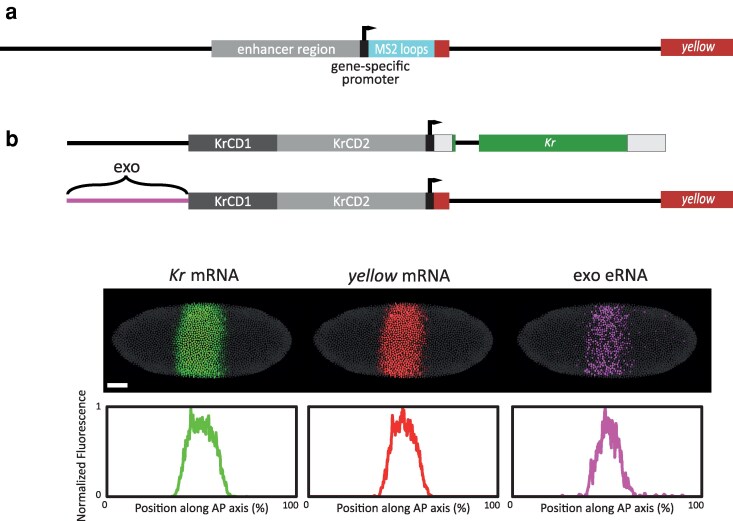
eRNA activity extends to reporter gene vector sequences. a) Schematic of the enhancer reporter construct: enhancer regions were cloned into the pbPHi plasmid upstream of gene-specific promoters. All constructs contain the *yellow* gene with 24xMS2 stem loops for live imaging, and the mini-white marker for selection. Site-specific integration was achieved via PhiC31 integrase. b) Transcriptional profile of the *Kr* enhancer reporter containing KrCD1 and KrCD2 enhancers: *In situ* HCR staining of whole embryos (NC14) visualized *Kr* mRNA, *yellow* mRNA, and a bacterial exogenous (exo) sense eRNA. Anterior is left, dorsal is up. Scale bars: 50 µm.

This reporter system is based on the pbPHi vector, which contains the *Drosophila yellow* reporter gene with MS2 stem loops inserted into its 5′ untranslated region (UTR) and an attB site for site-specific genomic integration (Perry et al. 2010; Bothma et al. 2014). We cloned gap gene or pair-rule gene enhancers, along with their corresponding endogenous promoter sequences, into this construct to assay eRNA transcription (Fig. 2a). This system allows for the analysis of eRNA and *yellow* mRNA expression dynamics in fixed embryos using FISH, as well as in live embryos via the MS2-MCP system (Garcia et al. 2013). The MS2-MCP system enables real-time visualization of nascent transcription: male flies carrying the enhancer > MS2-yellow construct were crossed with females expressing a ubiquitously driven MS2 coat protein (MCP)-GFP fusion. In cells where the enhancer is active, the MS2-yellow transcript is produced and bound by MCP-GFP, resulting in localized GFP enrichment at sites of active transcription.

Using PhiC31 integrase-mediated site-specific transgenesis, we generated several transgenic *Drosophila* lines carrying enhancer region > MS2-yellow constructs. To validate this system, we utilized the previously characterized 6 kb upstream sequence of the *Kr* locus containing the KrCD1 and KrCD2 enhancers (KrCD1 + 2 reporter line). To distinguish eRNA transcripts generated by the transgenic construct from those produced at the endogenous *Kr* locus, we designed *in situ* HCR probes targeting an *Escherichia coli* (*E. coli*) derived exogenous (exo) sequence integrated with the reporter construct. Notably, also in this situation, eRNA's were found upstream of the KrCD1 enhancer, in the *E. coli* derived exo vector sequences, and they spatially correlated with mRNA transcripts from the KrCD1 + 2 reporter (Fig. 2b) as well. This shows that eRNAs can be generated in foreign bacterial sequences when these are brought close to an active enhancer. Thus, the eRNAs upstream of the KrCD1 enhancer (Fig. 1e) likely were induced by these enhancers, and were not due to previously overlooked “weak” enhancers in the endogenous gene.

### Analysis of eRNA transcription in the absence of a gene promoter

We used the *eve* enhancers as study case, as these enhancers do not produce detectable eRNA at the endogenous *eve* locus (Supplementary Fig. 3) enabling the analysis of eRNA transcription in a controlled context, free from endogenous eRNA.

To determine whether eRNA transcription merely reflects background transcriptional noise at the gene promoter, we engineered an enhancer reporter system utilizing the stripe-specific *even-skipped* (*eve*) enhancer 1 + 5 (+6,616 bp to +8,210 bp of the *eve* transcription start), which drives expression in stripes 1 and 5 of the characteristic seven-striped *eve* gene pattern (Fujioka et al. 1999). Notably, this enhancer does not produce detectable eRNA at the endogenous *eve* locus (Supplementary Fig. 3a), enabling us to analyze eRNA transcripts originating exclusively from the reporter construct without interference from endogenous eRNA.

**Fig. 3.**
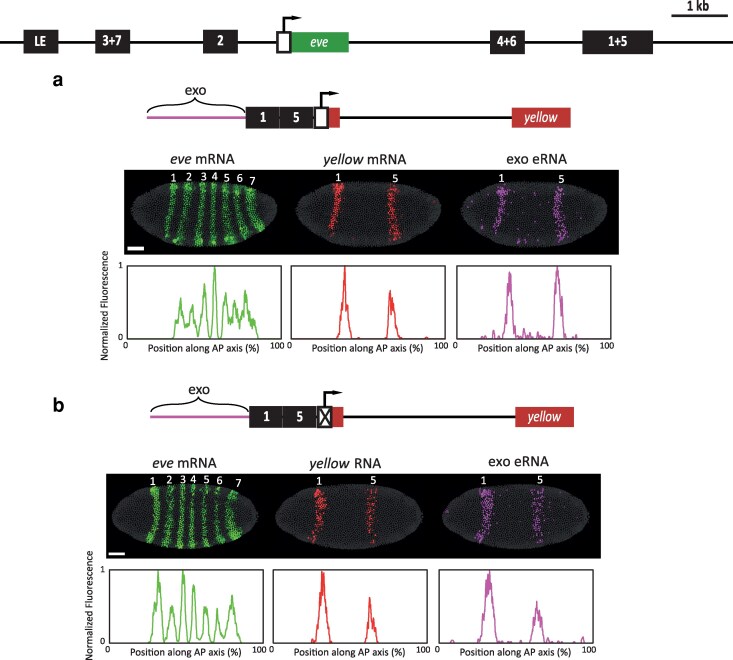
eRNA expression in the presence and absence of a promoter. *In situ* HCR staining (NC14) visualized mRNA (green/red) and sense eRNA (purple) together in whole embryos. Nuclei are gray. Fluorescence along the dorsal–ventral axis was integrated and normalized to generate AP distribution plots. a) eve1&5 enhancer reporter: the stripe-specific *eve* enhancer 1 + 5 drives *yellow* mRNA and eRNA at stripes 1 and 5. *yellow* transcripts are nuclear and cytoplasmic localized. Anterior is left, dorsal is up. b) eve1&5wop enhancer reporter (wop: without *eve* promoter [−50 bp to +50 bp of the *eve* transcription start]): Also when the promoter is deleted, the same enhancer drives weaker *yellow* RNA and eRNA at stripes 1 and 5 independent from a promoter. *yellow* RNA transcripts are nuclear localized and no longer cytoplasmic resembling eRNA transcripts. Anterior is left, dorsal is up. LE: late element enhancer. Scale bar: 50 µm.

Consistent with findings from the KrCD1 + 2 enhancer reporter, the eve1 + 5 reporter (for representative raw image see Supplementary Fig. 1c) also exhibited eRNA expression from vector sequences adjacent to the stripe enhancers, and also these exo eRNAs coincided spatially with mRNA expression of the *yellow* reporter gene (Fig. 3a). While the *eve* enhancer 1 + 5 shows no detectable eRNA expression at the endogenous locus, eRNA transcription is observed in the reporter construct, which may be enabled or enhanced by as yet unknown features of the reporter construct.

In addition, we generated a modified enhancer reporter lacking the gene promoter (eve1 + 5wop; wop: without *eve* promoter, i.e. lacking the interval from −50 bp to +50 bp relative to the *eve* transcription start site). In this line, RNA transcription was still detected, indicating that eRNA transcription can occur independently of the gene promoter. Both, exo and *yellow* region transcripts, were observed (Fig. 3b). In the absence of a gene promoter, *yellow* transcripts were mainly nuclear localized and no longer cytoplasmic, resembling the transcriptional properties of eRNAs. Cytoplasmic expression was minimal, as is typical for other eRNA transcripts. Endogenous *eve* mRNA transcription was unaffected, since the gene promoter was only removed from the reporter construct, whereas the endogenous gene promoter was still intact.

In summary, these findings demonstrate that eRNA transcription can occur independently of promoter-driven transcription. As next step, we tested if eRNA activation by enhancers can be blocked by insulators.

### Analyzing the influence of insulators on eRNA transcription

To test the influence of insulators on eRNA transcription, we established a 3&7&2 enhancer reporter line. This construct contains a 3.6 kb region upstream of the *eve* transcription start site, which includes the stripe-specific *eve* enhancers 3 + 7 (−3,817 bp to −3,307 bp relative to the *eve* transcription start site) and 2 (−1,557 bp to −1,073 bp). The 3 + 7 enhancer drives expression at stripes 3 and 7 in the characteristic *eve* pattern (Small et al. 1996), while the enhancer 2 drives expression at stripe 2 (Small and Levine 1991). As these enhancers do not produce detectable eRNA at the endogenous *eve* locus (Supplementary Fig. 3b+c), our system enables the analysis of eRNA transcription in a controlled context, free from endogenous eRNA. We visualized both, mRNA and sense eRNA expression, using *in situ* HCR staining.

**Fig. 4.**
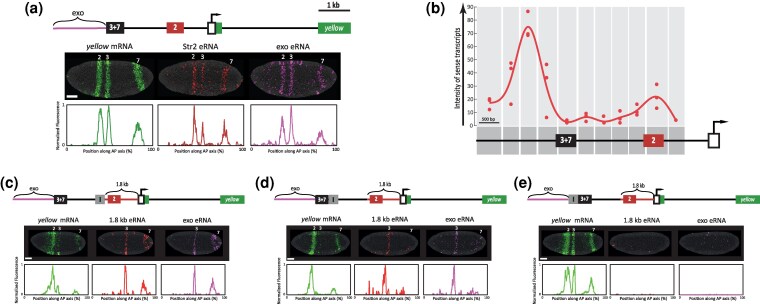
Influence of insulators on eRNA transcription. *In situ* HCR staining (NC14) visualized together mRNA (green) and sense eRNA (red/purple) in whole embryos. a) eve3&7&2 enhancer reporter: *yellow* mRNA, Str2 eRNA, and exo eRNA are detected at stripes 2, 3, and 7. b) Transcriptional profiling: A 5.5-kb upstream region (including endogenous enhancers and exogenous pbPHi sequence) was analyzed in 500-bp windows. The number of eRNA foci overlapping with *yellow* mRNA foci is plotted per embryo (*n* = 3). A maximum of eRNA is detected in the exogenous region, a small maximum just downstream of *eve* enhancer 3 + 7, and another one at *eve* enhancer 2. c–e) eRNA transcription blocked by su(Hw) insulator configurations: c) 3&7-su(Hw) upstream-2 transgene: Insulator is located 1.8 kb upstream of the *eve* transcription start site. *Yellow* mRNA is detected at stripe 2 and weakly at 3/7; eRNAs at stripes 3 and 7 only. d) 3&7-downstream su(Hw)-2 transgene: Insulator is positioned just downstream of enhancer 3 + 7. *Yellow* mRNA detected at stripes 2 and 7; eRNA at stripe 3 and weakly at 7. e) su(Hw)-3&7&2 transgene: Insulator was inserted upstream of enhancer 3 + 7. *Yellow* mRNA is expressed at stripes 2, 3, and 7; no eRNAs are detected. Anterior is left, dorsal is up. Scale bars: 50 µm.

The 3&7&2 enhancer reporter line exhibited eRNA expression at stripe positions 2, 3, and 7, coinciding with the *yellow* mRNA expression pattern (Fig. 4a). In line with the observation for eRNAs derived from the gt(−3) enhancer (Fig. 1d, gt(−3) eRNA), eve enhancer 2 showed eRNA expression at stripe positions 2,3, and 7, not only at stripe position 2 (Fig. 4a, Str2 eRNA).

Profiling the transcriptional landscape revealed maxima of eRNA expression at the exo region, a small maximum just downstream of *eve* enhancer 3 + 7, and another one at *eve* enhancer 2 (Fig. 4b). Reminiscent of our observations at the endogenous *Kr* locus, local minima of eRNA transcription were detected at enhancer region 3 + 7 and near the promoter. Since the profile lacks a sharp rise followed by a gradual decline and instead displays multiple maxima and minima, the existence of a cryptic promoter can be ruled out. We compared the eRNA transcription landscape profile we obtained with publicly available genomic datasets (Supplementary Fig. 2c) and found a similar overall trend, as the peaks identified by our FISH-based approach overlapped with peaks from PRO-seq, Zld ChIP-seq, and ATAC-seq.

The global maximum at the exo region appears to represent a region of strong transcriptional activity responsible for eRNA synthesis. To test this, we inserted an insulator sequence at various positions within the 3&7&2 enhancer reporter construct to investigate the effect of insulators on eRNA transcription, while the unaffected enhancer region 2 served as internal control. We utilized a 347 bp fragment containing su(Hw) binding sites (hereafter “su(Hw) insulator”), derived from the gypsy retrotransposon (Geyer and Corces 1992), which is well characterized for its ability to block enhancer–promoter communication in *Drosophila* (Cai and Levine 1995). Three insulator-containing reporter lines were generated: (i) su(Hw) insulator upstream of enhancer 2 (3&7-su(Hw) upstream-2), (ii) su(Hw) insulator downstream of enhancer 3 + 7 (3&7-downstream su(Hw)-2), and (iii) su(Hw) insulator upstream of enhancer 3 + 7 (su(Hw)-3&7&2). We then quantified mRNA transcripts from the exonic *yellow* region and eRNA transcripts from either the exo region or the 1.8 kb region upstream of the *eve* transcription start site.

In the 3&7-su(Hw) upstream-2 line, *yellow* mRNA was present at stripe 2 but only weakly at stripes 3 and 7. Residual mRNA at stripes 3 and 7 may result from incomplete insulation or from Pol II run-through, as these transcripts were predominantly nuclear and eRNA-like. Notably, *yellow* mRNA pattern at stripe 2 position was slightly weaker and not as concise as without the insulator (compare Fig. 4c to Fig. 4a, *yellow* mRNA) eRNA transcription was detected only at stripes 3 and 7, revealing the insulator's ability to block eRNA transcription (Fig. 4c), but seems to work different than with mRNA, since stripe 2 effect is blocked not only for exo eRNA but also for 1.8 kb eRNA.

Similarly, in the 3&7-downstream su(Hw)-2 line, eRNA transcription was blocked at stripe 2 (Fig. 4d). At stripe 7, eRNA expression was reduced while *yellow* mRNA expression increased, consistent with previous findings that *eve* expression at stripe 7 is driven not only by enhancer 3 + 7 but also by the intervening sequence between enhancers 3 + 7 and 2 (Small et al. 1996).

In the su(Hw)-3&7&2 line, eRNA transcription was completely abolished, while *yellow* mRNA was unaffected and expressed at stripes 2, 3, and 7 (Fig. 4e).

This shows that the spatial pattern of exo eRNAs is regulated by enhancers and can be blocked by insulators in a manner similar to enhancer–promoter interactions. However, the absence of eRNA transcripts at stripe position 2 for the 1.8 kb eRNA stainings remains unexplained, as this region should not be affected by the insulator—given that the *eve* enhancer 2 is not separated from the 1.8 kb region by it. One possible explanation is that the su(Hw) insulator exerts two distinct effects: (1) enhancer insulation, analogous to its function in blocking promoter–enhancer communication and (2) a stripe 2–specific silencing effect that operates in a position-independent manner.

Collectively, these insulator studies provide strong evidence that eRNA transcription is independently driven of gene promoters. This raises the question of whether, and if so, how eRNA transcription influences gene transcription itself. Our novel experimental approach could be used to address this question by leveraging the MS2-MCP live imaging capabilities of our enhancer reporter system.

### Promoter burst characteristics is influenced by the interaction of insulator and stripe 2

As a proof of concept that our constructs can be used for live imaging, and that one can use this tool for future analysis of eRNA function in live embryos, we investigated the influence of the insulator on stripe 2 activity by comparative live imaging on the 3&7&2 and the 3&7-su(Hw) upstream-2 enhancer reporter lines from early to late NC 14, using two-minute time intervals. The analysis focused specifically on stripe position 2, where mRNA expression is affected by a nearby insulator in an unexpected manner (Fig. 4c, *yellow* mRNA slightly weaker and less concise).

**Fig. 5.**
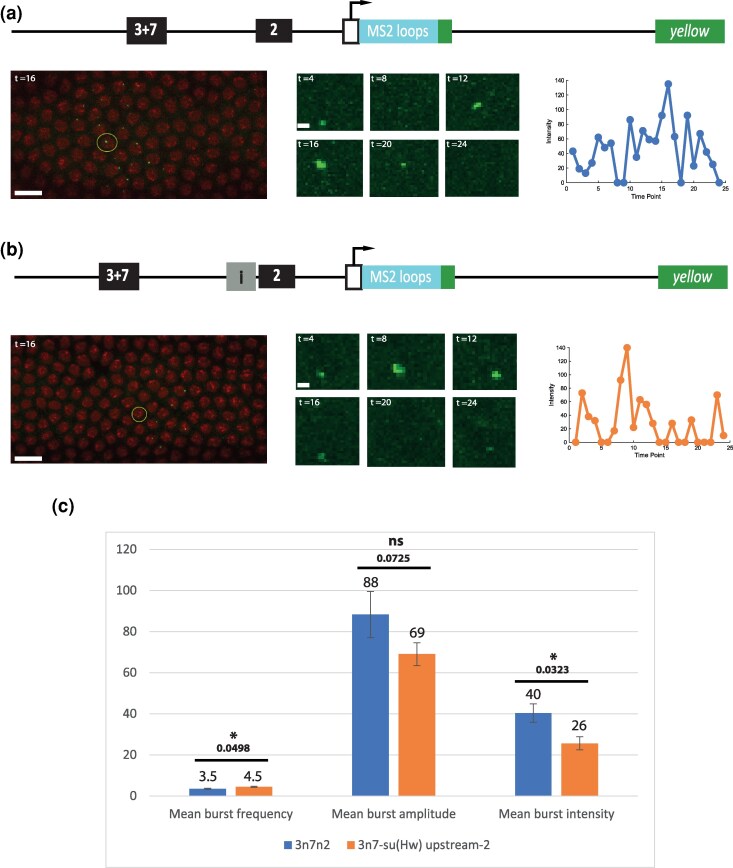
Analysis of the influence of an insulator on transcriptional burst activity at *eve* stripe 2. a, b) Live imaging of embryos expressing “3&7&2 > MS2-*yellow*; MCP-GFP; Histone-RFP” a) and “3&7-su(Hw) upstream-2 > MS2-*yellow*; MCP-GFP; Histone-RFP” b) from early to late NC 14 provides time-lapse visualization (2-min intervals) of active transcriptional sites, with nuclei marked in red (Histone-RFP) and nascent transcripts visualized as green GFP puncta. Yellow circles indicate analyzed nuclei, and anterior is to the left (scale bars: 10 µm). For each condition, representative nuclei traces show transcriptional bursting over time (puncta snapshots, scale bars: 1 µm). c) Quantitative comparison reveals a significant increase in mean burst frequency (bursts were identified as periods when the signal exceeds 5% of the maximum trace intensity, 4.5 ± 0.25 vs 3.5 ± 0.25, *P* = 0.0498) and a significant decrease in mean burst intensity (average signal across the entire trace, 26 ± 3.24 vs 40 ± 4.45, *P* = 0.0323), but no significant difference in mean burst amplitude (average of the maximum signal intensities within each burst, 69 ± 5.58 vs 88 ± 11.20, *P* = 0.0725) in the 3&7-su(Hw) upstream-2 reporter relative to 3&7&2 (*n* = 4 nuclei in one embryo per reporter line).

In the 3&7&2 reporter, prominent GFP puncta were evident at stripe position 2, signifying active transcription sites that mirrored the *yellow* mRNA expression pattern seen in HCR stainings (Supplementary Video 1, Fig. 5a). In the 3&7-su(Hw) upstream-2 line, where the insulator blocks eRNA expression at stripe position 2 and partially inhibits mRNA expression (Fig. 4c), GFP puncta were also visible, but slightly weaker (Supplementary Video 2, Fig. 5b).

Live imaging analysis of 4 nuclei in one embryo per reporter line revealed a statistically significant increase in mean transcriptional burst frequency, where bursts were identified as periods when the signal exceeds 5% of the maximum trace intensity, in the 3&7-su(Hw) upstream-2 line (4.5 ± 0.2450) compared to the 3&7&2 line (3.5 ± 0.2500; *P* = 0.0498). The mean burst amplitude (average of the maximum signal intensities within each burst), while slightly reduced in the 3&7-su(Hw) upstream-2 line (69 ± 5.58) vs the 3&7&2 line (88 ± 11.20), did not reach statistical significance (*P* = 0.0725). Importantly, mean burst intensity (average signal across the entire trace) was significantly lower in the 3&7-su(Hw) upstream-2 line (26 ± 3.24) relative to the 3&7&2 reporter (40 ± 4.45; *P* = 0.0323) (Fig. 5c).

These subtle shifts could reflect direct insulator effects on chromatin architecture or local Pol II availability, indirect consequences of blocked eRNA transcription, or a combination of both. While eRNA suppression might deplete a local Pol II pool and prompt compensatory increases in burst frequency, the modest magnitude of changes precludes definitive attribution to any single mechanism.

## Discussion

Enhancer RNAs (eRNAs) have been identified across a broad range of organisms—including *Drosophila*, *Caenorhabditis elegans*, mice, and humans (Ashe et al. 1997; Kim et al. 2010; Chen et al. 2013; Meers et al. 2018)—highlighting their potential as important regulators of transcription. In this study, we established an imaging-based framework to investigate eRNA transcriptional dynamics at the single-cell level in a controlled system. By integrating high-sensitivity HCR staining, high-resolution confocal microscopy, and enhancer reporter assays, we elucidated the characteristics of eRNA transcription in whole fixed or live *Drosophila* embryos.

Our findings provide new insights into the spatial and temporal dynamics of eRNAs and their functional relation to enhancers. We observed that eRNAs are expressed in similar temporal and spatial patterns as their associated enhancers (Fig. 1a to d). While temporal co-activity between eRNAs and enhancers had already been reported (Mikhaylichenko et al. 2018; Gao et al. 2023), the overlap in spatial expression patterns has so far been largely assumed. Our data therefore strengthen this link and support the idea that eRNAs can serve as reliable reporters of enhancer activity in both time and space.

Interestingly, we detected eRNA transcription not only within canonical enhancer regions but also in nearby sequences (Fig. 1e), challenging the prevailing view that eRNAs originate strictly from enhancer regions (Cheng et al. 2015; Franco et al. 2018). Instead, our results indicate that eRNAs can be produced in the vicinity of active enhancers. Moreover, we found bi-directional eRNA transcription in the endogenous *Kr* locus detected in line with previous studies on divergent transcription at *Drosophila* enhancers (Mikhaylichenko et al. 2018; Rennie et al. 2018). Critically, this eRNA production occurs independently of any promoter presence (Fig. 3), underscoring that enhancers alone are sufficient to drive non-coding transcription in their vicinity.

eRNA transcriptional activity can be effectively blocked by the introduction of an insulator element (Fig. 4c), suggesting that local chromatin architecture plays a critical role in defining the boundaries of eRNA activity. Moreover, eRNA transcription that can be blocked by insulators suggests that the sources of eRNA transcription may possess promoter-like characteristics. This idea aligns with previous studies showing that many active enhancers initiate local transcription through factors and mechanisms similar to those used by canonical promoters (Tippens et al. 2018). Genome-wide studies using techniques such as CAGE and GRO-seq have revealed that enhancers and promoters share similar chromatin marks (e.g. H3K27ac, H3K4me1/3), transcription factor occupancy, and RNA Pol II recruitment, and that both can serve as sites of divergent transcription initiation (Tippens et al. 2018; Kristjánsdóttir et al. 2020).

Even more strikingly, we observed eRNA production in unrelated bacterial sequences when these were positioned near active enhancers (Fig. 2b). This finding indicates that eRNA transcription is not limited to endogenous enhancer-associated sequences and can be induced simply by proximity to an active enhancer. Therefore, the presence of eRNAs in such regions cannot be explained by overlooked weak enhancers in the endogenous genome but rather reflects a broader property of enhancer-driven transcription.

We also identified a potential insulator effect on a downstream enhancer that could arise through blocking eRNAs transcribed from upstream regions (Fig. 5). This observation raises the intriguing possibility that eRNAs themselves may contribute to enhancer interference or insulation effects, suggesting a more complex and dynamic regulatory interplay between neighboring enhancers.

We hypothesize that RNA Polymerase II (Pol II) clusters arrive at the gene locus in an episodic manner. Upon arrival, Pol II molecules are distributed in the vicinity of active enhancers. Regions of eRNA transcription might act as reservoirs, storing Pol II around the gene locus and facilitating its recycling to the gene promoter when needed, thereby supporting sustained mRNA transcription. This model suggests that transcriptional bursting is modulated by the frequency of Pol II cluster recruitment. A previous study suggests, that Pol II transcription at non-coding regions suppresses transcriptional bursting from linked genes (Hamamoto et al. 2023). Nevertheless, more in detail analysis of live imaging constructs with other enhancers and insulators has to be performed to develop a universally valid model of the role of eRNA transcription in gene regulation.

Despite these observations, several questions remain. The precise molecular mechanisms by which eRNA transcription modulates Pol II dynamics and/or chromatin architecture are still unresolved, however it is suggested that Pol II is paused and released at enhancers and that histone H3 Lys4 (H3K4) methylation is present at actively transcribed enhancers (Henriques et al. 2018). Additionally, while our findings hint at the significance of eRNA transcription during early embryonic development, it is important to determine whether similar mechanisms operate in other tissues and developmental stages. Exact transcription start sites of eRNA transcripts could be determined using RACE or other unrelated sequences such as λDNA could be tested for eRNA transcription. In addition, insertion of polyA signals or depletion of components of the integrator complex could be done to test their influence on eRNA transcription.

The observations in this study are a proof of concept, that our FISH-based approaches is suited to study eRNA transcription in whole embryos. Here, we used detection windows of 500 bp, which could be further reduced to 100 bp resembling mapping resolution of genome-wide datasets. Moreover, our approach could be used in the future to do special transcriptomics on a single-cell level by increasing the number of analyzed transcripts through a sequential FISH technique recently developed by some of the authors (Garcia-Guillen et al. 2026).

Finally, our reporter vector system proved highly useful for dissecting enhancer–eRNA interactions and could be adapted for wider applications. In particular, it provides a versatile tool to experimentally test enhancer activity, insulator mechanisms, and non-coding transcription in diverse genomic contexts.

## Supplementary Material

jkag159_Supplementary_Data

## Data Availability

Primer sequences to generate constructs for transgenic lines are listed in Supplementary Table 1. LOT numbers of used HCR probes are listed in Supplementary Table 2. Code is available in Supplementary File 1. Program 1 was used to detect transcription sites in Figs. 1c to e, 2b, 3, and 4. Program 2 was used to create AP and DV plots in Figs. 1c+1d, 2b, 3, and 4a+4c to e. Program 3 was used to generate eRNA transcription profiles in Figs. 1e and 4b. Program 4 was used for burst analysis in Fig. 5. Supplementary Videos 1 and 2 are available on the G3 FigShare portal at https://doi.org/10.25387/g3.32685525. Supplemental material available at G3 online.
