# Supplementary material for: Enhancer RNA transcription near segmentation gene enhancers can be analyzed in situ using FISH
Source: G3 (Bethesda). 2026 Jun 19;16(8):jkag159. doi: 10.1093/g3journal/jkag159 (PMC13439931; doi:10.1093/g3journal/jkag159)
Supplement: Supplementary file 1 [file jkag159_supplementary_data.zip › Figure_S1_G3-2026-406907.pdf]

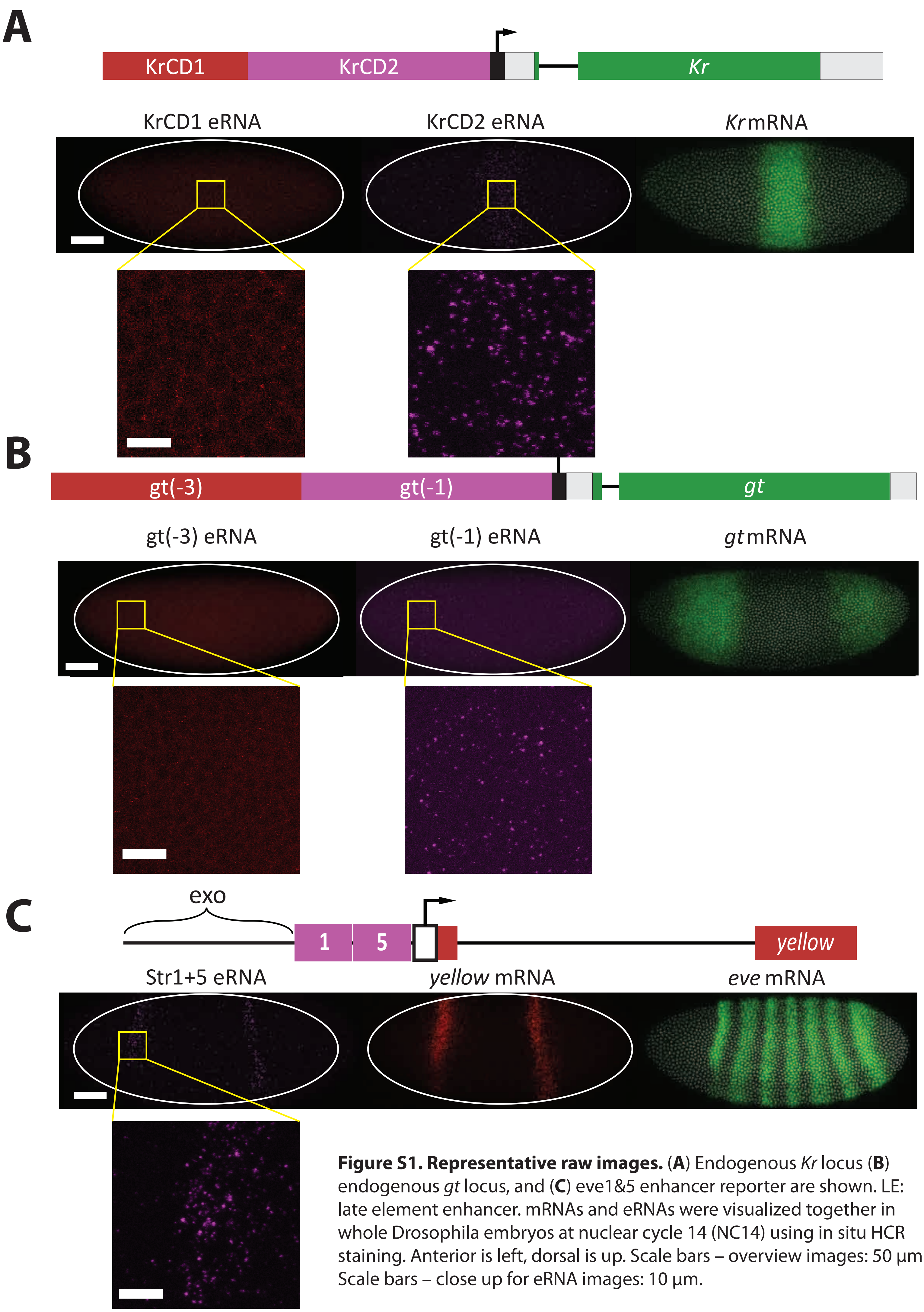

Figure S1. Representative raw images. (**A**) Endogenous *Kr* locus (**B**) endogenous *gt* locus, and (**C**) eve1&5 enhancer reporter are shown. LE: late element enhancer. mRNAs and eRNAs were visualized together in whole Drosophila embryos at nuclear cycle 14 (NC14) using in situ HCR staining. Anterior is left, dorsal is up. Scale bars – overview images: 50 µm. Scale bars – close up for eRNA images: 10 µm.
