# Supplementary material for: Enhancer RNA transcription near segmentation gene enhancers can be analyzed in situ using FISH
Source: G3 (Bethesda). 2026 Jun 19;16(8):jkag159. doi: 10.1093/g3journal/jkag159 (PMC13439931; doi:10.1093/g3journal/jkag159)
Supplement: Supplementary file 1 [file jkag159_supplementary_data.zip › Figure_S2_G3-2026-406907.pdf]

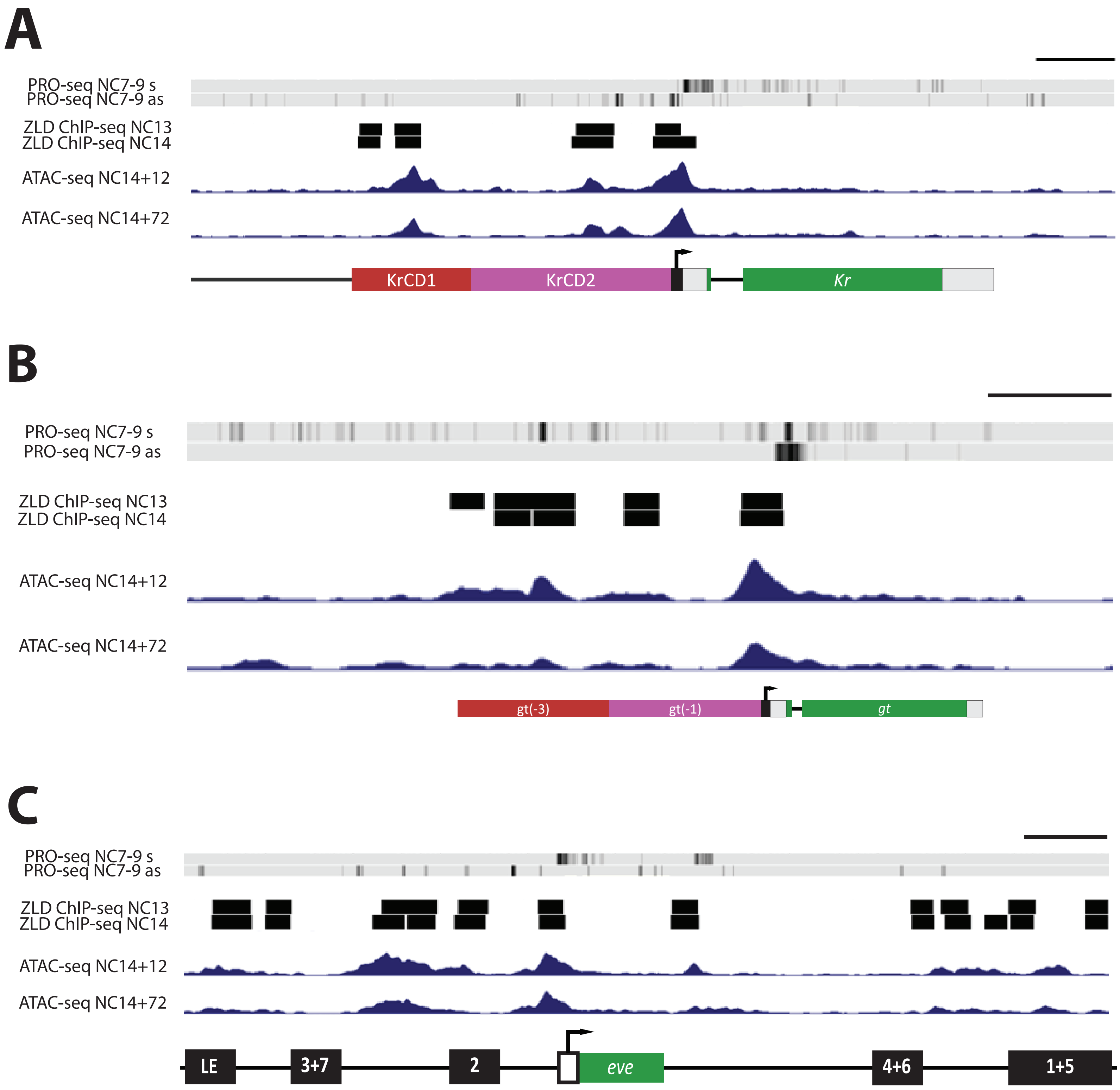

Figure S2. PRO-seq, ChIP-seq, and ATAC-seq data for *Kr*, *gt*, and *eve* locus. Shown are publicly available genomic datasets for the endogenous (**A**) *Kr*, (**B**) *gt*, and (**C**) *eve* locus. PRO-seq data (GEO accession: GSM6454906; Hunt et al., 2024) is obtained from embryos at NC7-9 (60-80 min after egg lay (AEL)) to visualize regions of active transcription (s: sense direction; as: anti-sense direction). ChIP-seq data (GEO accession: GSE30757; Harrison et al. ,2011) is obtained from embryos at NC13 (2h AEL) and NC14 (3h AEL) to visualize regions, where the pioneer factor Zelda (ZLD) needed for zygotic genome activation is bound. ATAC-seq data (GEO accession: GSE141538; Soluri et al., 2020) is obtained from embryos 12 min after the onset of NC14 (NC14+12) or 72 min after the onset of NC14 (NC14+72) to visualize open chromatin regions to identify regions that might be susceptible for eRNA transcription. Peaks of these genomic datasets coincide with found FISH signals. Tracks were visualized using the UCSC Genome Browser (Casper et al., 2026). Scale bars: 1 kb.
