# Supplementary material for: Enhancer RNA transcription near segmentation gene enhancers can be analyzed in situ using FISH
Source: G3 (Bethesda). 2026 Jun 19;16(8):jkag159. doi: 10.1093/g3journal/jkag159 (PMC13439931; doi:10.1093/g3journal/jkag159)
Supplement: Supplementary file 1 [file jkag159_supplementary_data.zip › Figure_S3_G3-2026-406907.pdf]

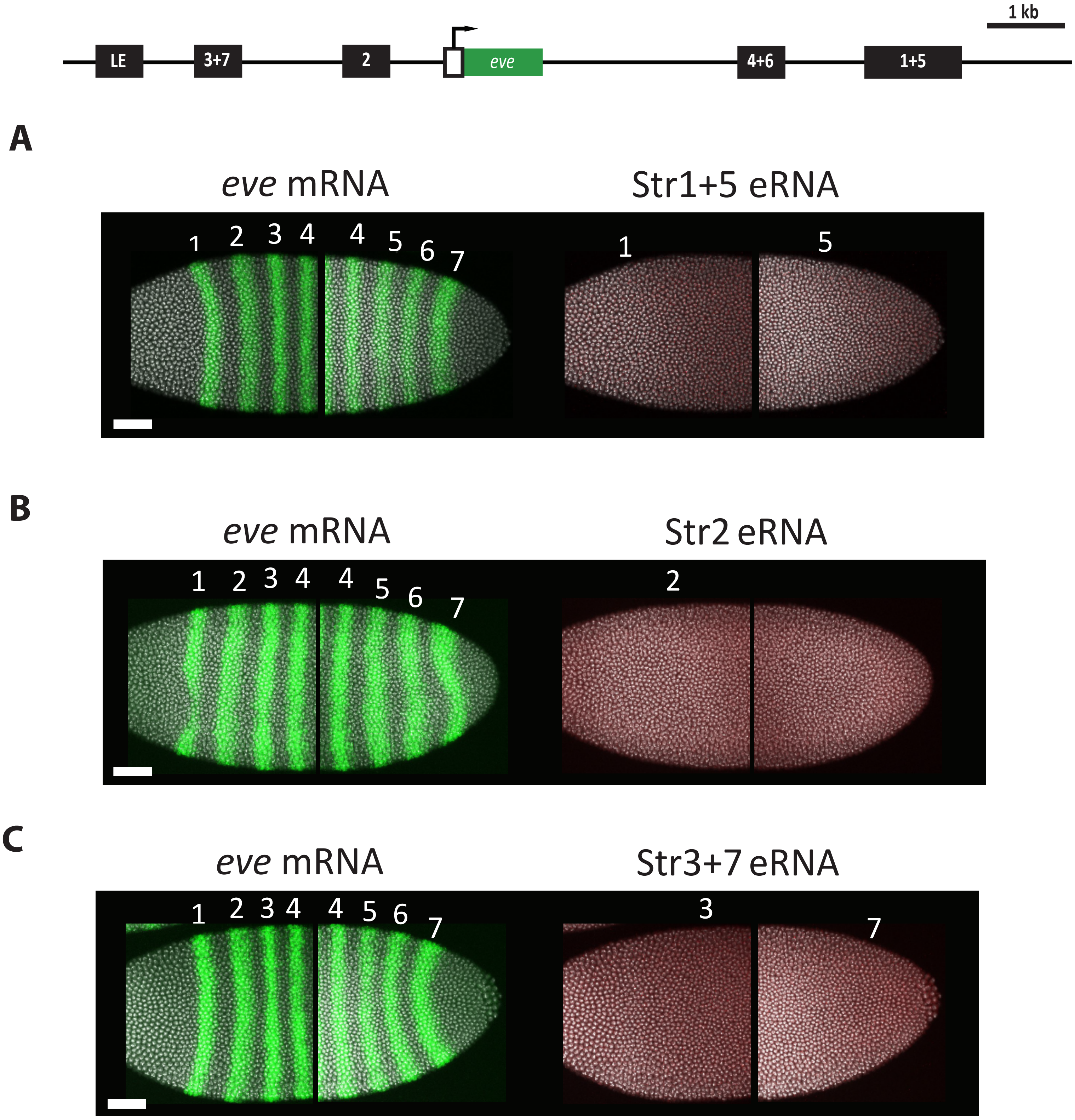

Figure S3. Spatial expression of eRNAs from *eve* enhancers at endogenous locus. mRNA (green) and eRNA (red) transcripts were visualized together in the endogenous *eve* locus in whole *Drosophila* embryos at nuclear cycle 14 (NC14) using *in situ* HCR staining. Nuclei are shown in grey. (**A**) *eve* 1+5 enhancer, (**B**) *eve* 2 enhancer, and (**C**) *eve* 3+7 enhancer showed no detectable eRNA transcripts. Anterior is left, dorsal is up. Scale bars: 50 µm.
