# Supplementary material for: Enhancer RNA transcription near segmentation gene enhancers can be analyzed in situ using FISH
Source: G3 (Bethesda). 2026 Jun 19;16(8):jkag159. doi: 10.1093/g3journal/jkag159 (PMC13439931; doi:10.1093/g3journal/jkag159)
Supplement: Supplementary file 1 [file jkag159_supplementary_data.zip › File_S1_G3-2026-406907.pdf]

File S1 – Fiji and Matlab code

1 Transcription site detection using Fiji

```
var nuclei_gaussian_blur = 5; // was 5 ; 15
var nuclei_prominence = 1; // was 1  (20x) ; 5 (63x)


function correctLabels() {
	setBatchMode("hide");


	getRawStatistics(nPixels, mean, min, nlabels, std, histogram);
	run("Duplicate...", "title=labels-corrected");
	run("Grays");
	run("Select All");
	setBackgroundColor(0, 0, 0);
	run("Clear", "slice");


	for(label = 1; label <= nlabels; label++) {
		selectWindow("labels");


		setThreshold(label, label);
		run("Create Selection");
		if(selectionType() == -1)
			continue;
		getSelectionBounds(x, y, w, h);
		run("Duplicate...", "title=label-tmp");
		run("Grays");
		setColor(65535);
		fill();
		run("Canvas Size...", "width=" + (3 * w) + " height=" + (3 * h) + " position=Center 
zero");
		run("Gaussian Blur...", "sigma=5");
		setThreshold(65535/2, 65535);
		run("Create Selection");
		if(selectionType() != -1) {
```

```
				getSelectionBounds(x2, y2, w2, h2);
				dx = x2 - w;
				dy = y2 - h;

				selectWindow("labels-corrected");
				run("Restore Selection");
				Roi.move(x + dx, y + dy);

				setColor(label);
				fill();
			}
			close("labels-keepLabels");
			showProgress(label, nlabels);
		}
		selectWindow("labels");
		run("Select None");
		setBatchMode("show");
		selectWindow("labels-corrected");
		run("Select None");
		resetMinAndMax();
		run("glasbey");
		setBatchMode("show");
		setBatchMode(false);
}

function segmentNucleus() {
		run("Duplicate...", "title=tmp duplicate channels=1");
		if(nSlices > 1)
			run("Z Project...", "projection=[Max Intensity]");
		rename("zprojection");
		close("tmp");
```

```
	run("Duplicate...", "title=[after max]");
	run("Enhance Local Contrast (CLAHE)", "blocksize=128 histogram=256 maximum=3 
mask=*None* fast_(less_accurate)");
	getRawStatistics(nPixels, mean, min, max, std, histogram);
	run("Max...", "value=" + (max / 2));
	rename("after max");

	run("Duplicate...", "title=[for find maxima]");
	run("Gaussian Blur...", "sigma=" + nuclei_gaussian_blur);
	run("Find Maxima...", "prominence=" + nuclei_prominence + " output=[Single Points]");
	rename("seeds");
	close("for find maxima");

	selectWindow("after max");
	run("Duplicate...", "title=[for watershed]");
	run("Gaussian Blur...", "sigma=5");
	run("OICE Watershed", "seeds=seeds");
	rename("watershed");
	run("Duplicate...", "title=labels-without-background");
	resetMinAndMax();
	run("glasbey");
	save(folder + "/" + file + "-nulclei-without-background.tif");
	rename("labels-without-background");
	close("seeds");
	close("for watershed");

	selectWindow("after max");
	run("Gaussian Blur...", "sigma=1");
	setAutoThreshold("Otsu dark");
	setOption("BlackBackground", true);
	run("Convert to Mask");
	rename("mask");
```

```
		selectWindow("mask");
		run("16-bit");
		run("Divide...", "value=255");
		imageCalculator("Multiply", "watershed","mask");
		selectWindow("watershed");
		run("glasbey");
		rename("labels");
		close("mask");

		correctLabels();
		save(folder + "/" + file + "-nulclei-with-background.tif");

		close();
		close("labels");
		close("zprojection");
		/*
		label = getTitle();
		run("Label Boundaries");
		run("16-bit");
		run("Merge Channels...", "c1=[zprojection] c2=[" + label + "-bnd] create");
		rename("z projection");
		*/
}

parameterString = "";

folder = getInfo("image.directory");
file = getInfo("image.filename");
file = substring(file, 0, lastIndexOf(file, "."));

title = getTitle();
```

```
Stack.setChannel(1);
segmentNucleus();
parameterString = parameterString +
	"nuclei_gaussian_blur = " + nuclei_gaussian_blur + "\n" +
	"nuclei_prominence = " + nuclei_prominence + "\n";

selectWindow(title);
Stack.setChannel(2);
run("Select None");
call("lm.Plugin.setDefaultParameters", "0.5", "10", "1");
//call("lm.Plugin.setDefaultParameters", "0.5", "8", "1");
run("Local Maxima 3D");
parameterString = parameterString +
	"channel2_particle_size = " + call("lm.Plugin.getDefaultParticleSize") + "\n" +
	"channel2_threshold = "    + call("lm.Plugin.getDefaultLowerThreshold") + "\n" +
	"channel2_tolerance = "    + call("lm.Plugin.getDefaultTolerance") + "\n";
selectWindow("labels-without-background");
Table.applyMacro("NucleusID = getPixel(x__pixels_, y__pixels_);");
saveAs("Results", folder + "/" + file + "-channel2.csv");
selectWindow("Results");
run("Close");

selectWindow(title);
Stack.setChannel(3);
run("Select None");
call("lm.Plugin.setDefaultParameters", "0.5", "20", "1");
run("Local Maxima 3D");
parameterString = parameterString +
	"channel3_particle_size = " + call("lm.Plugin.getDefaultParticleSize") + "\n" +
	"channel3_threshold = "    + call("lm.Plugin.getDefaultLowerThreshold") + "\n" +
	"channel3_tolerance = "    + call("lm.Plugin.getDefaultTolerance") + "\n";
selectWindow("labels-without-background");
```

```
Table.applyMacro("NucleusID = getPixel(x__pixels_, y__pixels_);");
saveAs("Results", folder + "/" + file + "-channel3.csv");
selectWindow("Results");
run("Close");

selectWindow(title);
Stack.getDimensions(width, height, channels, slices, frames);
if(channels > 3) {
	selectWindow(title);
	Stack.setChannel(4);
	run("Select None");
	call("lm.Plugin.setDefaultParameters", "0.5", "5", "1");
	run("Local Maxima 3D");
	parameterString = parameterString +
		"channel4_particle_size = " + call("lm.Plugin.getDefaultParticleSize") + "\n" +
		"channel4_threshold = "     + call("lm.Plugin.getDefaultLowerThreshold") + "\n" +
		"channel4_tolerance = "     + call("lm.Plugin.getDefaultTolerance") + "\n" + "Tx_plane
= 0,1000" + "\n"+ "Loc = A" + "\n"+ "NC = 13";
	selectWindow("labels-without-background");
	Table.applyMacro("NucleusID = getPixel(x__pixels_, y__pixels_);");
	saveAs("Results", folder + "/" + file + "-channel4.csv");
	selectWindow("Results");
	run("Close");
}

File.saveString(parameterString, folder + "/parameters.txt");

close("labels-without-background");
Overlay.remove();
run("Select None");
```

2 Generation of AP and DV plots using Matlab

```
clear
clc
close all


parent_folder_name='parent_folder_name';
image_id='image_id';


voxel_dimensions=[0.08 0.08 0.44];
AP_location=1;
embryo= ExtractPuncta_SingleFile(parent_folder_name,image_id,AP_location);


puncta=embryo.puncta;
Lnuc=embryo.niceLnuc;
N_nuc=max(max(Lnuc));
S=embryo.size;
Nx=S(1); Ny=S(2);
if(length(S)>2)

    Nz=S(3);
else
    Nz=1;
end
Nc=embryo.number_of_channels;
Np=length(puncta);
N_puncta=cell(Np,1);
for n=1:Np
    N_puncta{n}=length(puncta{n});
end

Inuc_mask=double(Lnuc);
Inuc_mask(Inuc_mask>0)=255;


% puncta_intensity_thresholds=[50,20,20];
puncta_images_mapping=[1 2 3];%puncta type to image index



%Initiating nuclei list
for n=1:Np
    for m=N_nuc:-1:1
        Nuclei(m).puncta{n}=[];
        Nuclei(m).puncta{n}=[];
        Nuclei(m).puncta{n}=[];
    end
end

count_add=0;
[D,idx_nuc]=bwdist(imbinarize(Lnuc,0));


%adding punta intensities to corresponding nuclei
for n=1:Np
    for m=1:N_puncta{n}
```

```
        loc=puncta{n}(m).location;
        intensity=puncta{n}(m).intensity;

        nuc_id=Lnuc(loc(1),loc(2));

        if(nuc_id==0)
            nuc_id=find_nearest_label_to_point(loc(1:2),Lnuc,idx_nuc);
        end

        puncta{n}(m).nucleus_id=nuc_id;
        Nuclei(nuc_id).puncta{n}=[Nuclei(nuc_id).puncta{n} intensity];
    end
end



%making images with integrated intensity for each nucleus
I_integ=cell(3,1);
for n=1:Np
    I_integ{n}=zeros(Ny,Nx);
end

for m=1:N_nuc
    if(mod(m,100)==0)
        m
    end
    for n=1:Np

        SS=sum(Nuclei(m).puncta{n});


        I_integ{n}(Lnuc==m)=SS;
    end
end

figure
imshow(I_integ{1}/max(max(I_integ{1})).*double(Inuc_mask)/255*2)
saveas(gcf,'figure1.png')
figure
imshow(I_integ{2}/max(max(I_integ{2})).*double(Inuc_mask)/255*2)
saveas(gcf,'figure2.png')
figure
imshow(I_integ{3}/max(max(I_integ{3})).*double(Inuc_mask)/255*2)
saveas(gcf,'figure3.png')

Inuc=Lnuc;
Inuc(Inuc>1)=1;


Inuc_mask_clean=double(Inuc_mask)/255;


n=1;
mix=2;
Icolor=zeros(Ny,Nx,3);
Icolor(:,:,1)=0*mix*I_integ{n}/(max(max(I_integ{n}))).*Inuc_mask_clean+0.2*Inuc_ma
sk_clean; %10* for mrna
Icolor(:,:,2)=1*mix*I_integ{n}/(max(max(I_integ{n}))).*Inuc_mask_clean+0.2*Inuc_ma
sk_clean;
```

```
Icolor(:,:,3)=0*mix*I_integ{n}/(max(max(I_integ{n}))).*Inuc_mask_clean+0.2*Inuc_ma
sk_clean;
figure
imshow(Icolor)
saveas(gcf,'figure4.png')


n=2;
mix=2;
Icolor=zeros(Ny,Nx,3);
Icolor(:,:,1)=1*mix*I_integ{n}/(max(max(I_integ{n}))).*Inuc_mask_clean+0.2*Inuc_ma
sk_clean; %10* for mrna
Icolor(:,:,2)=0*mix*I_integ{n}/(max(max(I_integ{n}))).*Inuc_mask_clean+0.2*Inuc_ma
sk_clean;
Icolor(:,:,3)=0*mix*I_integ{n}/(max(max(I_integ{n}))).*Inuc_mask_clean+0.2*Inuc_ma
sk_clean;
figure
imshow(Icolor)
saveas(gcf,'figure5.png')


n=3;
mix=2;
Icolor=zeros(Ny,Nx,3);
Icolor(:,:,1)=1*mix*I_integ{n}/(max(max(I_integ{n}))).*Inuc_mask_clean+0.2*Inuc_ma
sk_clean; %10* for mrna
Icolor(:,:,2)=0*mix*I_integ{n}/(max(max(I_integ{n}))).*Inuc_mask_clean+0.2*Inuc_ma
sk_clean;
Icolor(:,:,3)=1*mix*I_integ{n}/(max(max(I_integ{n}))).*Inuc_mask_clean+0.2*Inuc_ma
sk_clean;
figure
imshow(Icolor)
saveas(gcf,'figure6.png')


% average across DV
figure
set(gca,'Color','w')
hold on
direction=1;%1 or 2
plot(mean(I_integ{1}*3,direction),'g','LineWidth',2)
saveas(gcf,'figure7.pdf')

figure
set(gca,'Color','w')
hold on
direction=1;%1 or 2
plot(mean(I_integ{2}*4,direction),'r','LineWidth',2)
saveas(gcf,'figure8.pdf')

figure
set(gca,'Color','w')
hold on
direction=1;%1 or 2
plot(mean(I_integ{3}*2,direction)*1,'m','LineWidth',2)
saveas(gcf,'figure9.pdf')


function embryo= ExtractPuncta_SingleFile(parent_folder_name,image_id,AP_location)
```

```
genomic_location = str2double(extractBetween(image_id,"No ","."));
if(isnan(genomic_location))
    genomic_location = str2double(extractBetween(image_id,"No "," -"));
end


folder_name=[parent_folder_name image_id '\'];
raw_image_fname = [folder_name image_id];


TiffData = Tiff([raw_image_fname '.tif'],'r');
tagValue = getTag(TiffData,'ImageDescription');
%number of channels
Nc = str2double(extractBetween(string(tagValue),"channels=",string(newline)));
%number of puncta to analyze
Np=Nc-1;
%size of z stack
Nz = str2double(extractBetween(string(tagValue),"slices=",string(newline)));
% size of x-dimension (loc(2))
Nx=getTag(TiffData,'ImageWidth');
% size of y-dimension (loc(1))
Ny=getTag(TiffData,'ImageLength');


%reading file in to cell array image I
I=cell(Nc,1);
for nc=1:Nc
    I{nc}=zeros(Ny,Nx,Nz);
end
k=0;
for nz=1:Nz
    for nc=1:Nc
        k=k+1;
        I{nc}(:,:,nz)=imread([raw_image_fname '.tif'], k);
    end
end

%nuclear mask for computation (voronoi)
nuclear_mask_fname= [raw_image_fname  '-nulclei-without-background.tif'];
Lnuc=imread(nuclear_mask_fname);
N_nuc=max(max(Lnuc));


%nuclear mask for visualization (separate individual nuclei)
nice_nuclear_mask_fname= [raw_image_fname  '-nulclei-with-background.tif'];
niceLnuc=imread(nice_nuclear_mask_fname);
niceInuc=niceLnuc;
niceInuc(niceInuc>1)=1;



puncta=cell(Np,1);
puncta_th=cell(Np,1);
puncta_intensities=cell(Np,1);
puncta_images_mapping=[2 3 4];%[3 4 2];%puncta type to image index
N_puncta=cell(Np,1);

puncta_table_names=cell(Np,1);
```

```
for np=1:Np
    puncta_table_names{np}= [raw_image_fname '-channel' int2str(np) '.csv'];
end


params_fname = [folder_name 'parameters.txt'];
params_text = fileread(params_fname);
particle_sizes = zeros(1,Nc-1);
thresholds = zeros(1,Nc-1);
tolerances = zeros(1,Nc-1);

% example: Tx_plane = 9,19
for nc=1:Nc-1
    particle_sizes(nc)=str2double(extractBetween(params_text,['channel'
int2str(nc+1) '_particle_size = '] ,string(newline)));
    thresholds(nc)=str2double(extractBetween(params_text,['channel' int2str(nc+1)
'_threshold = '] ,string(newline)));
    tolerances(nc)=str2double(extractBetween(params_text,['channel' int2str(nc+1)
'_tolerance = '] ,string(newline)));
end
Tx_plane=zeros(1,2);
Tx_plane(1)=str2double(extractBetween(params_text,'Tx_plane = ' ,','));
Tx_plane(2)=str2double(extractAfter(params_text,','));


thresholds =[4,8,6];
% thresholds =[2,5];

%extracting puncta
tic
warning off
for n=1:Np
    puncta_table_name= [raw_image_fname '-channel' int2str(n+1) '.csv'];
    puncta{n}=
extract_puncta_ver8_FromTableANDImage(I{puncta_images_mapping(n)},puncta_table_nam
e,thresholds(n));
    N_puncta{n}=length(puncta{n});
end
warning on
toc

embryo.puncta= puncta;
embryo.Lnuc= Lnuc;
embryo.niceLnuc= niceLnuc;
embryo.size= [Nx Ny Nz];
embryo.number_of_channels=Nc;
embryo.detection_params.particle_sizes=particle_sizes;
embryo.detection_params.thresholds=thresholds;
embryo.detection_params.tolerances=tolerances;
embryo.Tx_plane=Tx_plane;
embryo.AP_location=AP_location;
embryo.genomic_location=genomic_location;


function array_puncta=
extract_puncta_ver8_FromTableANDImage(I,table_file_name,intensity_thresh)

%load table
T = readtable(table_file_name);
```

```
%read puncta locations and convolved intensities
puncta_locs=[T.x_pixels_, T.y_pixels_, T.z_pixels_];
puncta_locs_micron=[T.x_micron_, T.y_micron_, T.z_micron_];

puncta_intensities=T.peakIntensity_convolved_;
nucleus_id=T.NucleusID;

puncta_locs(puncta_intensities<intensity_thresh,:)=[];
puncta_intensities(puncta_intensities<intensity_thresh,:)=[];

%number of pucta
N=size(puncta_locs,1);


punctum= struct(...
    'location',[],... %x,y,zpuncta_intensities{n}(m)
    'intensity',[],...
    'volume',0,...
    'nucleus_id',[]);

array_puncta=repmat(punctum,N,1);


for n=1:N

    nx=puncta_locs(n,2)+1;
    ny=puncta_locs(n,1)+1;
    nz=puncta_locs(n,3)+1;

    nx_micron=puncta_locs_micron(n,2);
    ny_micron=puncta_locs_micron(n,1);
    nz_micron=puncta_locs_micron(n,3);


    array_puncta(n).location=[nx ny nz];
    array_puncta(n).location_micron=[nx_micron ny_micron nz_micron];

    array_puncta(n).intensity=puncta_intensities(n);%intensity;
    array_puncta(n).nucleus_id=nucleus_id(n);


end


function nearest_label=find_nearest_label_to_point(point,L,D)

index_to_closest_point=D(point(1),point(2));
nearest_label=L(index_to_closest_point);
```

3 Generation of eRNA transcription profiles using Matlab

Extract transcription site for a batch of embryos

```
clear
clc

parent_folder='parent_folder';
% Get a list of all files and folders in this folder.
files = dir(parent_folder);
% Get a logical vector that tells which is a directory.
dirFlags = [files.isdir];
% Extract only those that are directories.
subFolders = files(dirFlags);
subFolders=subFolders(3:end);

%save puncta analysis folders and embryo overview folders in separate
%variables: punctaFolders and overviewFolders
punctaFolders=subFolders;
overviewFolders=subFolders;
punctaFolders_toDelete=[];
overviewFolders_toDelete=[];
for n=1:length(subFolders)
    if(contains(subFolders(n).name,"overview"))
        punctaFolders_toDelete=[punctaFolders_toDelete n];
    else
        overviewFolders_toDelete=[overviewFolders_toDelete n];
    end
end
punctaFolders(punctaFolders_toDelete)=[];
overviewFolders(overviewFolders_toDelete)=[];

%read embryo orientations
embryoOrientations=zeros(length(punctaFolders),1);
for n=1:length(overviewFolders)
    orientation_fname=[parent_folder overviewFolders(n).name '\Orientation.txt'];
    orientation_text = fileread(orientation_fname);
    if(orientation_text=='AP')
        embryoOrientations(2*(n-1)+1)=1;
        embryoOrientations(2*(n-1)+2)=2;
    else
        embryoOrientations(2*(n-1)+1)=2;
        embryoOrientations(2*(n-1)+2)=1;
    end

end

allEmbryos=cell(length(punctaFolders),1);
counter=0;
allAttributesMatrices=[];
for n=1:length(punctaFolders)

embryo=ExtractPuncta_Single(parent_folder,punctaFolders(n).name,embryoOrientations
(n));
    embryo.embryo_id=ceil(n/2);
    save([parent_folder punctaFolders(n).name '\embryo.mat'],'embryo')
    allEmbryos{n}=embryo;
```

```
    Attributes_Matrix=Generate_Attributes_Matrix(embryo);
    save([parent_folder punctaFolders(n).name
'\Attributes_Matrix.mat'],'Attributes_Matrix')



Attributes_Matrix(Attributes_Matrix(:,11)>0,11)=Attributes_Matrix(Attributes_Matri
x(:,11)>0,11)+size(allAttributesMatrices,1);

Attributes_Matrix(Attributes_Matrix(:,12)>0,12)=Attributes_Matrix(Attributes_Matri
x(:,12)>0,12)+size(allAttributesMatrices,1);

Attributes_Matrix(Attributes_Matrix(:,13)>0,13)=Attributes_Matrix(Attributes_Matri
x(:,13)>0,13)+size(allAttributesMatrices,1);
    allAttributesMatrices=[allAttributesMatrices; Attributes_Matrix];

    counter=counter+size(Attributes_Matrix,1);

%     input('ddd:')
end


save([parent_folder '\allEmbryos.mat'],'allEmbryos', '-v7.3')
save([parent_folder '\allAttributesMatrices.mat'],'allAttributesMatrices')

n=1;
mean(allAttributesMatrices(allAttributesMatrices(:,2)==2 &
allAttributesMatrices(:,13)>0 & allAttributesMatrices(:,1)==n &
allAttributesMatrices(:,4)==1,8))
mean(allAttributesMatrices(allAttributesMatrices(:,2)==2 &
allAttributesMatrices(:,13)>0 & allAttributesMatrices(:,1)==n &
allAttributesMatrices(:,4)==2,8))
function Attributes_Matrix=Generate_Attributes_Matrix(embryo)

puncta=embryo.puncta;

total_RNAs=embryo.total_RNAs;


Nc=length(puncta);%number of channels

N_attributes=16;

Np=zeros(1,Nc);%number of puncta in each channel

for np=1:Nc
    Np(np)=size(puncta{np},1);
end

% Attributes
% (01) embryo #
% (02) channel
% (03) genomic_location
% (04) location in the embryo (Anterior:1, posterior:2)
% (05) x location
% (06) y location
% (07) z location
% (08) intensity
% (09) nuc_id
```

```
% (10) Nnuc
% (11) index of overlapping channel 1
% (12) index of overlapping channel 2
% (13) index of overlapping channel 3
% (14) total RNA in channel 1 within host nucleus
% (15) total RNA in channel 2 within host nucleus
% (16) total RNA in channel 3 within host nucleus

%%%%%%%%%%%%%%%%%%%%%%%%%%%% calculate overlappings %%%%%%%%%%%%%%%%%

randomized_flag=0;
puncta_intensity_thresholds=[0*2200,0*2200,0*240];
z_th_lower=embryo.Tx_plane(1);
z_th_upper=embryo.Tx_plane(2);
%vertical
y_th_lower=0;
y_th_upper=inf;
%horizontal
x_th_lower=0;
x_th_upper=inf;

if(randomized_flag)
    for n=Np:-1:1
        outls_puncta{n}= shuffle_puncta_locations(puncta{n},Lnuc);
    end
else
    outls_puncta=puncta;
end


for np=1:Nc
    m_to_delete=[];
    for m=1:length(outls_puncta{np})

        if(outls_puncta{np}(m).intensity<puncta_intensity_thresholds(np)...
                || outls_puncta{np}(m).location(3)<z_th_lower ||
outls_puncta{np}(m).location(3)>z_th_upper...
                || outls_puncta{np}(m).location(2)<x_th_lower ||
outls_puncta{np}(m).location(2)>x_th_upper...
                || outls_puncta{np}(m).location(1)<y_th_lower ||
outls_puncta{np}(m).location(1)>y_th_upper)

            m_to_delete=[m_to_delete m];
        end


    end
    outls_puncta{np}(m_to_delete)=[];
end

for np=1:Nc
    Np(np)=size(outls_puncta{np},1);
end
Attributes_Matrix=zeros(sum(Np),N_attributes);


voxel_dimensions=[0.08 0.08 0.44];
th_distance=8*voxel_dimensions(1);%8%5%10
```

```
AB= detect_two_overlapping_puncta(outls_puncta{2},outls_puncta{3},
voxel_dimensions, th_distance);

%%%%%%%%%%%%%%%%%%%%%%%%%%%%%%%%%%%%%%%%%%%%%%%%%%%%%%%%%%%%%


% Fill Attributes Matrix
count=0;
for np=1:Nc

    for n=1:Np(np)
        count=count+1;

        punctum=outls_puncta{np}(n);
        loc=punctum.location;

        Attributes_Matrix(count,1)=embryo.embryo_id;
        Attributes_Matrix(count,2)=np;
        Attributes_Matrix(count,3)=embryo.genomic_location;
        Attributes_Matrix(count,4)=embryo.AP_location;
        Attributes_Matrix(count,5)=loc(1);
        Attributes_Matrix(count,6)=loc(2);
        Attributes_Matrix(count,7)=loc(3);
        Attributes_Matrix(count,8)=punctum.intensity;
        Attributes_Matrix(count,9)=punctum.nucleus_id;
        Attributes_Matrix(count,10)=max(max(embryo.Lnuc));

        % MODIFY: develop a tie breaker
        AB_1_address=find(AB(:,1)==n);
        M1=length(AB_1_address);
        AB_2_address=find(AB(:,2)==n);
        M2=length(AB_2_address);
        if(np==2 && ~isempty(AB_1_address))
            for m1=1:M1
                Attributes_Matrix(count,13)=AB(AB_1_address(m1),2)+Np(1)+Np(2);
            end
        elseif(np==3 && ~isempty(AB_2_address))
            for m2=1:M2
                Attributes_Matrix(count,12)=AB(AB_2_address(m2),1)+Np(1);
            end
        end



        Attributes_Matrix(count,14)=total_RNAs(1,punctum.nucleus_id);
        Attributes_Matrix(count,15)=total_RNAs(2,punctum.nucleus_id);
        Attributes_Matrix(count,16)=total_RNAs(3,punctum.nucleus_id);



    end

end


function embryo= ExtractPuncta_Single(parent_folder_name,image_id,AP_location)


genomic_location = str2double(extractBetween(image_id,"No","."));
```

```
if(isnan(genomic_location))
    genomic_location = str2double(extractBetween(image_id,"No","-"));
end

% image_id
genomic_location
% input('zeo:')

folder_name=[parent_folder_name image_id '\'];
raw_image_fname = [folder_name image_id];


TiffData = Tiff([raw_image_fname '.tif'],'r');
tagValue = getTag(TiffData,'ImageDescription');
%number of channels
Nc = str2double(extractBetween(string(tagValue),"channels=",string(newline)));
%number of puncta to analyze
Np=Nc-1;
%size of z stack
Nz = str2double(extractBetween(string(tagValue),"slices=",string(newline)));
% size of x-dimension (loc(2))
Nx=getTag(TiffData,'ImageWidth');
% size of y-dimension (loc(1))
Ny=getTag(TiffData,'ImageLength');


%reading file in to cell array image I
I=cell(Nc,1);
for nc=1:Nc
    I{nc}=zeros(Ny,Nx,Nz);
end
k=0;
for nz=1:Nz
    for nc=1:Nc
        k=k+1;
        I{nc}(:,:,nz)=imread([raw_image_fname '.tif'], k);
    end
end

%nuclear mask for computation (voronoi)
nuclear_mask_fname= [raw_image_fname  '-nulclei-without-background.tif'];
Lnuc=imread(nuclear_mask_fname);
N_nuc=max(max(Lnuc));


%nuclear mask for visualization (separate individual nuclei)
nice_nuclear_mask_fname= [raw_image_fname  '-nulclei-with-background.tif'];
niceLnuc=imread(nice_nuclear_mask_fname);
niceInuc=niceLnuc;
niceInuc(niceInuc>1)=1;



puncta=cell(Np,1);
puncta_th=cell(Np,1);
puncta_intensities=cell(Np,1);
puncta_images_mapping=[2 3 4];%[3 4 2];%puncta type to image index
N_puncta=cell(Np,1);
```

```
puncta_table_names=cell(Np,1);
for np=1:Np
    puncta_table_names{np}= [raw_image_fname '-channel' int2str(np) '.csv'];
end



params_fname = [folder_name 'parameters.txt'];
params_text = fileread(params_fname);
particle_sizes = zeros(1,Nc-1);
thresholds = zeros(1,Nc-1);
tolerances = zeros(1,Nc-1);



% example: Tx_plane = 9,19
for nc=1:Nc-1
    particle_sizes(nc)=str2double(extractBetween(params_text,['channel'
int2str(nc+1) '_particle_size = '] ,string(newline)));
    thresholds(nc)=str2double(extractBetween(params_text,['channel' int2str(nc+1)
'_threshold = '] ,string(newline)));
    tolerances(nc)=str2double(extractBetween(params_text,['channel' int2str(nc+1)
'_tolerance = '] ,string(newline)));
end
Tx_plane=zeros(1,2);
Tx_plane(1)=str2double(extractBetween(params_text,'Tx_plane = ' ,',','));
Tx_plane(2)=str2double(extractAfter(params_text,','));


thresholds =[4,2,2];%[15    60];


%extracting puncta
tic
warning off
for n=1:Np
    puncta_table_name= [raw_image_fname '-channel' int2str(n+1) '.csv'];
    puncta{n}=
extract_puncta_ver8_FromTableANDImage(I{puncta_images_mapping(n)},puncta_table_nam
e,thresholds(n));
    N_puncta{n}=length(puncta{n});
end
warning on
toc



%total flueorescence lists: total_RNAs
total_RNAs=zeros(Np,N_nuc);
for np=1:Np
    for n=1:length(puncta{np})
        nucleus_id=puncta{np}(n).nucleus_id;

        if(nucleus_id>N_nuc)
            image_id
            N_nuc
            nucleus_id
        end
```

```
total_RNAs(np,nucleus_id)=total_RNAs(np,nucleus_id)+puncta{np}(n).intensity;
    end
end

%total fluorescence images: I_RNA
%NOTE: use command 'impixelinfo' to displace pixel values
I_RNA=cell(Np,1);
for np=1:Np
    I_RNA{np}=zeros(Ny,Nx);
    for nuc=1:N_nuc
        I_RNA{np}(Lnuc==nuc)=total_RNAs(np,nuc);
    end
end


embryo.puncta= puncta;
embryo.Lnuc= Lnuc;
embryo.niceLnuc= niceLnuc;
embryo.total_RNAs=total_RNAs;
embryo.I_RNA=I_RNA;
embryo.size= [Nx Ny Nz];
embryo.number_of_channels=Nc;
embryo.detection_params.particle_sizes=particle_sizes;
embryo.detection_params.thresholds=thresholds;
embryo.detection_params.tolerances=tolerances;
embryo.Tx_plane=Tx_plane;
embryo.AP_location=AP_location;
embryo.genomic_location=genomic_location;




function array_puncta=
extract_puncta_ver8_FromTableANDImage(I,table_file_name,intensity_thresh)



%load table
T = readtable(table_file_name);


%read puncta locations and convolved intensities
puncta_locs=[T.x_pixels_, T.y_pixels_, T.z_pixels_];
puncta_locs_micron=[T.x_micron_, T.y_micron_, T.z_micron_];

puncta_intensities=T.peakIntensity_convolved_;
nucleus_id=T.NucleusID;

puncta_locs(puncta_intensities<intensity_thresh,:)=[];
puncta_intensities(puncta_intensities<intensity_thresh,:)=[];

%number of pucta
N=size(puncta_locs,1);


punctum= struct(...
    'location',[],... %x,y,zpuncta_intensities{n}(m)
    'intensity',[],...
```

```
    'volume',0,...
    'nucleus_id',[]);

array_puncta=repmat(punctum,N,1);


for n=1:N

    nx=puncta_locs(n,2)+1;
    ny=puncta_locs(n,1)+1;
    nz=puncta_locs(n,3)+1;

    nx_micron=puncta_locs_micron(n,2);
    ny_micron=puncta_locs_micron(n,1);
    nz_micron=puncta_locs_micron(n,3);

    array_puncta(n).location=[nx ny nz];
    array_puncta(n).location_micron=[nx_micron ny_micron nz_micron];

    array_puncta(n).intensity=puncta_intensities(n);%intensity;
    array_puncta(n).nucleus_id=nucleus_id(n);


end


function [AB]= detect_two_overlapping_puncta(A_puncta,B_puncta, voxel_dimensions,
th_distance)

Na= length(A_puncta);
Nb= length(B_puncta);


%connectivity matrices
AB=[];

%Finding AB overlappings
for na=1:Na
    if(mod(na,100)==0)
        [num2str(na) ' out of ' num2str(Na)]
    end

    for nb=1:Nb

        Dab=sqrt(sum(((A_puncta(na).location-
B_puncta(nb).location).*voxel_dimensions).^2));

        if(Dab<th_distance)

                AB=[AB; na nb];
        end

    end


end

AB=unique(AB,'rows');
```

Visualization of spatial eRNA profile

```
clear
clc
close all

parent_folder='parent_folder';


% load([parent_folder '\allEmbryos.mat'],'allEmbryos')
load([parent_folder '\allAttributesMatrices.mat'],'allAttributesMatrices')

genomic_loci=178:189;
scaled_genomic_loci=movmean([0,.0385,.12,.2115,.308,.393,.4695,.557,.6435,.7255,.8
075,.8845,.9615,1],2);
scaled_genomic_loci=scaled_genomic_loci(2:end-1);


channel=2;
TS_ch2_thresh=20;
loc=1;
GenomeWalk_y=[];
GenomeWalk_x=[];
for n=1:length(genomic_loci)
    G=allAttributesMatrices(allAttributesMatrices(:,3)==genomic_loci(n) ...
        & allAttributesMatrices(:,4)==loc & allAttributesMatrices(:,2)==channel
...
        & allAttributesMatrices(:,8)>TS_ch2_thresh,8);



    G_embryos=allAttributesMatrices(allAttributesMatrices(:,3)==genomic_loci(n)
...
        & allAttributesMatrices(:,4)==loc & allAttributesMatrices(:,2)==channel
...
        & allAttributesMatrices(:,8)>TS_ch2_thresh,1);

    embryos=unique(G_embryos);
    % length(embryos)


    for m=1:length(embryos)
        number_of_TSs(m,n)=length(G(G_embryos==embryos(m)));
        avgIntensity_of_TSs(m,n)=mean(G(G_embryos==embryos(m)));
        totalIntensity_of_TSs(m,n)=sum(G(G_embryos==embryos(m)));
        % number_of_TSs
    end

end


figure
f=fit(scaled_genomic_loci',mean(number_of_TSs,1)','smoothingspline','SmoothingPara
m',1);
plot(f, 'b')
hold on
for m=1:2
    scatter(scaled_genomic_loci',number_of_TSs(m,:)','b.')
end
```

```
figure
f=fit(scaled_genomic_loci',mean(avgIntensity_of_TSs,1)','smoothingspline','Smoothi
ngParam',.5);
plot(f)
hold on
for m=1:2
    scatter(scaled_genomic_loci',avgIntensity_of_TSs(m,:)','b.')
end
figure
f=fit(scaled_genomic_loci',mean(totalIntensity_of_TSs,1)','smoothingspline','Smoot
hingParam',1);
plot(f)
hold on
for m=1:2
    scatter(scaled_genomic_loci',totalIntensity_of_TSs(m,:)','b.')
end
```

4 Bursting analysis using Matlab

```
clear
clc

% Data input
data = { ...
    [% data]
...
};
labels = {% labels};
n_nuclei = numel(data);

% Preallocate results
burst_freq = zeros(1,n_nuclei);
burst_amp = zeros(1,n_nuclei);
mean_intensity = zeros(1,n_nuclei);



% Analysis
for i=1:n_nuclei
    trace = data{i};
    thresh = 0.05 * max(trace); % Define threshold for burst, 5% of the max trace
intensity
    mean_intensity(i) = mean(trace);
    % Find bursts: simple threshold crossing
    above_thresh = trace > thresh;
    d_bt = diff([0 above_thresh 0]);
    burst_starts = find(d_bt == 1);
    burst_ends = find(d_bt == -1) - 1;
    num_bursts = length(burst_starts);
    burst_freq(i) = num_bursts / length(trace); % Burst per time point
    if num_bursts > 0
        amp_list = zeros(1,num_bursts);
        for b = 1:num_bursts
            amp_list(b) = max(trace(burst_starts(b):burst_ends(b)));
        end
        burst_amp(i) = mean(amp_list);
    else
        burst_amp(i) = NaN;
    end
end

% Display results
results_table = table(labels', burst_freq', burst_amp', mean_intensity', ...
    'VariableNames', {'Nucleus','BurstFreq','BurstAmplitude','MeanIntensity'});
disp(results_table);

% Visual Representation

figure;
subplot(2,2,1)
bar(burst_freq)
set(gca,'XTickLabel',labels)
ylabel('Burst Frequency (per Tp)')
title('Burst Frequency')

subplot(2,2,2)
```

```
bar(burst_amp)
set(gca,'XTickLabel',labels)
ylabel('Burst Amplitude')
title('Burst Amplitude')

subplot(2,2,3)
bar(mean_intensity)
set(gca,'XTickLabel',labels)
ylabel('Mean Intensity')
title('Mean Intensity')

subplot(2,2,4)
hold on
colors = lines(n_nuclei);
for i=1:n_nuclei
    plot(data{i}, '-o', 'DisplayName',labels{i},'Color',colors(i,:))
end
% Calculate and plot mean trace
lengths = cellfun(@length,data);
maxlen = max(lengths);
data_padded = nan(n_nuclei,maxlen);
for i = 1:n_nuclei
    data_padded(i,1:length(data{i})) = data{i};
end
mean_trace = mean(data_padded, 1, 'omitnan');
plot(mean_trace, '--k', 'LineWidth',2, 'DisplayName','Mean')
xlabel('Time Point')
ylabel('Intensity')
title('Traces (Individual & Mean)')
legend show
hold off

% Calculate means across all nuclei
mean_burst_freq = mean(burst_freq, 'omitnan');
mean_burst_amp = mean(burst_amp, 'omitnan');
mean_intensity_all = mean(mean_intensity, 'omitnan');

% Display in command window
fprintf('Mean burst frequency: %.3f (per time point)\n', mean_burst_freq);
fprintf('Mean burst amplitude: %.3f\n', mean_burst_amp);
fprintf('Mean intensity: %.3f\n', mean_intensity_all);

% Add means to bar plots
figure;
subplot(2,2,1)
bar(burst_freq)
set(gca,'XTickLabel',labels)
ylabel('Burst Frequency (per Tp)')
title('Burst Frequency')
hold on
plot(xlim, [mean_burst_freq mean_burst_freq],'k--
','LineWidth',2,'DisplayName','Mean')
hold off

subplot(2,2,2)
bar(burst_amp)
set(gca,'XTickLabel',labels)
ylabel('Burst Amplitude')
title('Burst Amplitude')
```

```
hold on
plot(xlim, [mean_burst_amp mean_burst_amp],'k--
','LineWidth',2,'DisplayName','Mean')
hold off

subplot(2,2,3)
bar(mean_intensity)
set(gca,'XTickLabel',labels)
ylabel('Mean Intensity')
title('Mean Intensity')
hold on
plot(xlim, [mean_intensity_all mean_intensity_all],'k--
','LineWidth',2,'DisplayName','Mean')
hold off

subplot(2,2,4)
hold on
colors = lines(n_nuclei);
for i=1:n_nuclei
    plot(data{i}, '-o', 'DisplayName',labels{i},'Color',colors(i,:))
end
% Mean trace
maxlen = max(cellfun(@length,data));
data_mat = nan(n_nuclei, maxlen);
for i=1:n_nuclei
    data_mat(i,1:length(data{i})) = data{i};
end
mean_trace = mean(data_mat,1,'omitnan');
plot(mean_trace, 'k-', 'LineWidth', 2, 'DisplayName','Mean')
xlabel('Time Point')
ylabel('Intensity')
title('Traces (Individual & Mean)')
legend show
hold off
```
