# Supplementary material for: Enhancer RNA transcription near segmentation gene enhancers can be analyzed in situ using FISH
Source: G3 (Bethesda). 2026 Jun 19;16(8):jkag159. doi: 10.1093/g3journal/jkag159 (PMC13439931; doi:10.1093/g3journal/jkag159)
Supplement: Supplementary file 1 [file jkag159_supplementary_data.zip › Table_S1_G3-2026-406907.pdf]

Table S1. List of used primers. Frw: forward, rev: reverse.

Construct	Description	Sequence (5` - 3`)
KrCD1+2	KrCD1+2 frw	CAGGTCGCAGCGGCCGCTTAAACTATTTTGGAAACTCAAAT
	KrCD1+2 rev	AAGTACCGTAGGATCCGGCACAACAAATGGTTGTTCCT
eve1&5	1&5 frw	CAGGTCGCAGCGGCCGCGGCCTAATCACTTCCCTGAAATG
	1&5 rev	AGCAAGCTTGCGGCCGCAAATTGATTGCCCTGCGG
eve1&5wop	1&5wop frw	CAGGTCGCAGCGGCCGCGGCCTAATCACTTCCCTGAAATG
	1&5wop rev	AAGTACCGTAGGATCCAAATTGATTGCCCTGCGG
eve3&7&2	3&7&2 frw	CAGGTCGCAGCGGCCGCGGATCCTCGAAATCGAGAGCGAC
	3&7&2 rev	AAGTACCGTAGGATCCAACGAAGGCAGTTAGTTGTTGACTG
eve3&7-su(Hw) upstream-2	3&7 frw	AGATCCAGGTCGCAGCGGCCGCGGATCCTCGAAATCGAGAGCGAC
	3&7 rev	CGAATAAATTTTCTTGCCCCGGGAAAGGTTC
	su(Hw) upstream frw	GGGGCAAGAAAATTTATTCGCAAAAACATTGCA
	su(Hw) upstream rev	CATGCAAGCCTATGGCAAGAAAAGGTATGCAA
	2 frw	TCTTGCCATAGGCTTGCATGTGGGCCTT
	2 rev	GCAATAAGTACCGTAGGATCCAACGAAGGCAGTTAGTTGTTGACT
eve3&7-downstream su(Hw)-2	3&7 frw	AGATCCAGGTCGCAGCGGCCGCAGAGCGACCTCGCTGCAT
	3&7 rev	CGAATAAATTGAGCTCGTAAAAACGTGAATGCCA
	downstream su(Hw) frw	TTACGAGCTCAATTTATTCGCAAAAACATTGCA
	downstream su(Hw) rev	CCGAAGGAACTATGGCAAGAAAAGGTATGCAA
	2 frw	TCTTGCCATAGTTCCTTCGGGTCCAAAATTATGCC
	2 rev	GCAATAAGTACCGTAGGATCCAACGAAGGCAGTTAGTTGTTGACTG
su(Hw)-eve3&7&2	su(Hw) frw	AGATCCAGGTCGCAGCGGCCGCAATTTATTCGCAAAAACATTGCA
	su(Hw) rev	AGGTCGCTCTTATGGCAAGAAAAGGTATGCAA
	3&7&2 frw	TCTTGCCATAAGAGCGACCTCGCTGCAT
	3&7&2 rev	GCAATAAGTACCGTAGGATCCAACGAAGGCAGTTAGTTGTTGACTG
