# Supplementary material for: Enhancer RNA transcription near segmentation gene enhancers can be analyzed in situ using FISH
Source: G3 (Bethesda). 2026 Jun 19;16(8):jkag159. doi: 10.1093/g3journal/jkag159 (PMC13439931; doi:10.1093/g3journal/jkag159)
Supplement: Supplementary file 1 [file jkag159_supplementary_data.zip › Table_S2_G3-2026-406907.pdf]

Table S2. LOT numbers for HCR probe sets. Landscape profiling: individual probes of a probe set separately delivered allowing for eRNA transcription detection with a resolution down to 500 bp.

**LOT** Number	Target	HCR Amplifier
PRA987	*yellow* mRNA	B1
PRB336	*Kr* mRNA	B1
PRB337	KrCD1 sense eRNA	B2
PRB338	KrCD1 anti-sense eRNA	B2
PRB339	KrCD2 sense eRNA	B3
PRB340	KrCD2 anti-sense eRNA	B3
PRB341	*eve* mRNA	B1
PRB874	*eve* stripe3&7 sense eRNA - landscape profiling	B2
PRB875	*eve* intergenic region between *eve* stripe 3&7 and stripe 2 sense eRNA - landscape profiling	B2
PRB876	*eve* stripe2 sense eRNA - landscape profiling	B2
PRB877	*eve* intergenic region between *eve* stripe 2 and *eve* transcription start site sense eRNA - landscape profiling	B2
PRB883	*eve* stripe1 sense eRNA	B2
PRB884	*eve* stripe5 sense eRNA	B2
PRC021	*gt* mRNA	B3
PRC022	gt(-1) sense eRNA	B1
PRC023	gt(-1) anti-sense eRNA	B1
PRC024	gt(-3) sense eRNA	B2
PRC025	gt(-3) anti-sense eRNA	B2
PRC654	*yellow* mRNA	B2
PRC655	*yellow* mRNA	B3
PRC668	pBPhi exo sense eRNA	B3
PRD263	*Kr* locus sense eRNA - landscape profiling	B2
PRD266	*Kr* locus anti-sense eRNA - landscape profiling	B3
PRE719	pBPhi exo sense eRNA - landscape profiling	B2
